# Idiosyncratic Eye‐Movement Patterns to Faces Are Associated With Differences in Neural Processing: Evidence From Behavioral and Event‐Related Potential Responses

**DOI:** 10.1111/ejn.70726

**Published:** 2026-10-22

**Authors:** Nianzeng Zhong, Janet H. Hsiao, Antoni B. Chan, William G. Hayward

**Affiliations:** ^1^ Department of Psychology Lingnan University Hong Kong SAR; ^2^ Cognitive Science Research Centre Lingnan University Hong Kong SAR; ^3^ Division of Social Science Hong Kong University of Science & Technology Hong Kong SAR; ^4^ Department of Computer Science City University of Hong Kong Hong Kong SAR

**Keywords:** event‐related potentials (ERPs), eye movements, face identification, individual differences, N170, P1

## Abstract

Although faces are homogeneous stimuli, individuals adopt different eye‐movement patterns when looking at them. Do such differences in eye movements reflect distinct patterns of neural processing of faces? We conducted two experiments to investigate how face‐viewing preferences affect face‐sensitive event‐related potentials (ERPs). In both experiments, participants were separated into two groups: an upper‐focused group who fixated mainly on the eyes of faces and a lower‐focused group who fixated more on the nose and mouth. Participants then completed two fixation number‐controlled blocks in Experiment 1 or two fixation duration‐controlled blocks in Experiment 2, in which the initial fixation location was restricted to the left eye or the nose. In both fixation number‐controlled and duration‐controlled blocks, the upper‐focused group performed better and elicited a larger P1‐to‐N170 peak‐to‐peak amplitude for the eye‐fixation condition than the nose‐fixation condition. In contrast, the lower‐focused group performed evenly and elicited comparable P1‐to‐N170 amplitudes between fixation conditions. In addition, the P1 component was larger for the nose‐fixation condition than the eye‐fixation condition, and this difference was bigger for the lower‐focused group than the upper‐focused group. On the other hand, the P1 appeared to have no relation to behavioral performance. These findings show how individual differences in fixation patterns for faces map to differences in neural processing of those faces, and in turn provide evidence for the intricate relationship between sensory and neural processing of visual information.

## Graphical abstract

We manipulated fixation locations on faces during an identification task. The results demonstrated that participants who prefer to observe the eyes performed better and showed larger P1‐to‐N170 peak‐to‐peak amplitudes when fixating on the eyes than on the nose, whereas those who prefer to observe the center of faces showed no significant differences between fixation conditions. These results suggest that face identification performance and neural responses are associated with idiosyncratic eye‐movement patterns and the face region that is being fixated.

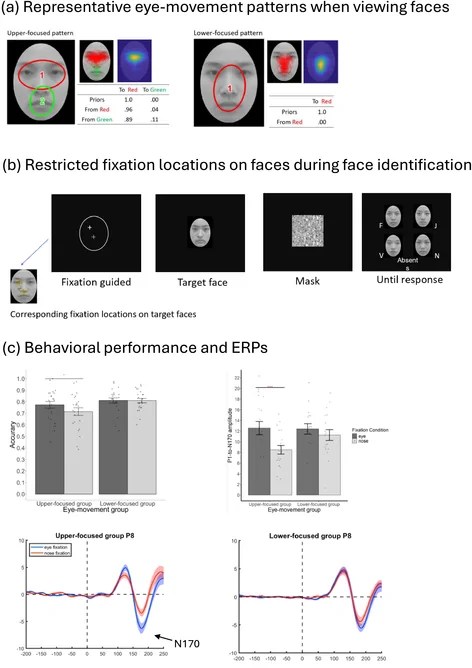

**Abbreviations**
EEG: electroencephalography
EM: eye‐movement
EMHMM: eye movement hidden Markov model
ERP: event‐related potential
HMM: hidden Markov Model
ROI: region‐of‐interest
SE: standard error

## 1 Introduction

Since Yarbus's seminal study using eye tracking techniques to measure observers' eye movements, many studies have found that people commonly fixate on the inner features of faces, such as the eyes, the nose, and the mouth, when viewing faces (e.g., Althoff and Cohen 1999; Caldara et al. 2010; Yarbus 1965). However, recent studies have revealed that these eye‐movement (EM) patterns differ between individuals when they are identifying faces. For example, some observers prefer to fixate on the upper regions of faces such as the eyes, although still showing a few fixations on the lower halves of faces (Chuk et al. 2014; Chuk, Crookes, et al. 2017; An and Hsiao 2021; Peterson and Eckstein 2012). In contrast, other observers prefer to fixate on features in the lower halves of faces, such as the nose, but generally avoid looking at the eyes. Thus, one key difference between individuals' EM patterns is along the vertical dimension of the face, with some observers fixating higher and others observing lower. Most individuals' EM patterns can be well‐represented by the division of an upper‐focused group and a lower‐focused group (Chuk et al. 2014; Peterson and Eckstein 2012, 2013; Zhong et al. 2024), although some studies have adopted more detailed divisions of EM patterns, such as including left‐eye‐biased pattern and right‐eye‐biased pattern (Arizpe et al. 2017; Chuk, Crookes, et al. 2017). In addition, these individual differences seem relatively stable; several studies have found that these fixation preferences are consistent across thousand trials in the lab (Peterson and Eckstein 2012, 2013; Tsank and Eckstein 2017), on that day and many days later (Mehoudar et al. 2014; Peterson and Eckstein 2012), from the lab to the real world (Peterson et al. 2016), and even to non‐face objects (Broda and de Haas 2024). However, despite the stability of these differences, little is known about the role that individuals' viewing preferences play in face identification or how they influence (or are influenced by) underlying neural mechanisms of face perception. In this paper, we investigate the association between fixation preferences and neural processing of a face.

Previous studies have suggested that individuals' looking preferences seek to optimize face identification performance. For example, Peterson and Eckstein (2012) found that individuals were worse at identifying faces when fixating on the forehead or mouth than when they fixated on the eyes or nose. However, viewing location affects identification performance unequally for upper‐focused and lower‐focused observers (Peterson and Eckstein 2013). Upper‐focused observers performed worse than lower‐focused observers when they were asked to fixate on the nose. In contrast, when they were required to fixate on the eyes, lower‐focused observers performed as well as upper‐focused observers. In addition, individuals' preferences affect holistic processing of faces, as measured by the composite effect. Zhong et al. (2024) tested participants on a composite task, which consisted of a top‐cued block in which they were asked to match the upper halves of two consecutive faces, and a bottom‐cued block in which the bottom halves were the targets. The upper‐focused group showed a larger composite effect on matching upper halves of faces than matching lower halves, whereas the lower‐focused group showed no difference in the size of the composite effect for matching upper halves and lower halves of faces. This result demonstrates that holistic processing varies with the match between preferred viewing location and actual viewing location. Since holistic processing has been considered one of the hallmarks of face processing (for a review, see Murphy et al. 2017; Rossion 2013), and the size of the composite effect has been found to correlate positively with face recognition (DeGutis et al. 2013; Wang et al. 2012; but see Konar et al. 2010; Richler et al. 2015), these findings imply that upper‐focused observers' identification performance may be sensitive to fixation location, whereas lower‐focused observers' performance may be relatively insensitive to fixation location.

In addition, some studies have examined the neural sensitivity to faces and fixation locations measured by electroencephalography (EEG). The N170, a characteristic negative deflection electrical event‐related potentials (ERPs) in the parieto‐occipital region that occurs at about 170 ms after stimulus onset, is a face‐sensitive component (for a review, see Eimer 2011; Yovel 2008). N170 amplitude is larger for faces than for objects such as cars (Bentin et al. 1996; Rossion and Caharel 2011), houses (Nemrodov et al. 2014), and watches (De Lissa et al. 2014). Moreover, the sensitivity of the N170 differs in fixation locations of faces. Using a fixation cross to guide participants to look at different locations, researchers found that N170 amplitudes were larger when the fixations were on the nasion and mouth than on the nose (McPartland et al. 2010). Using a more stringent control of fixations, researchers required participants to fixate on specific locations, and found that N170 amplitude was larger when the viewing location was the eyes as compared with when it was the forehead, nasion, nose, or mouth (Nemrodov et al. 2014). In another study using a similar approach, researchers also found larger P1‐to‐N170 peak‐to‐peak amplitudes when participants fixated on the eyes directly than when they fixated on the mouth (De Lissa et al. 2014). Overall, the N170 was larger for fixations on upper halves of faces, especially the eyes, than on lower halves of faces, such as the nose or mouth. Researchers proposed that the eyes are the most informative feature on a face, and fixating the eyes facilitates face perception at the early stages (Nemrodov et al. 2014). However, those studies grouped neural responses of all participants together but did not pay attention to idiosyncratic EM patterns. Since the eyes are the default viewing region for upper‐focused individuals but not for lower‐focused individuals, we did not know whether the larger N170 was elicited by fixating the eyes per se or by individuals' looking preference, and therefore, whether both EM groups would elicit a larger N170 when fixating on upper halves than lower halves of faces.

Furthermore, the P1, an early ERP component occurring about 100 ms after stimulus onset (Jeffreys and Axford 1972), has also been explored in previous studies about face fixations. However, the results of such studies are inconsistent. Some researchers found that P1 amplitude was not affected by viewing position of faces (De Lissa et al. 2014; Nemrodov et al. 2014); some others found that P1 amplitude was larger for fixations on the eyes than on the mouth (McPartland et al. 2010); and yet others found that P1 amplitude was lower with fixations on the eyes than on the nose (Peterson et al. 2014). Other studies have found that the P1 appears to be sensitive to early visual processing of low‐level visual properties such as luminance and contrast (Ganis et al. 2012; Johannes et al. 1995; Rossion and Caharel 2011), and P1 amplitude triggered by faces is similar to that triggered by scrambled images created from those faces (Rossion and Caharel 2011), indicating that these P1 amplitude effects might be related to low‐level properties instead of high‐level facial featural information at different fixation locations. Thus, we expected that the P1 would not be modulated by looking preferences or fixation locations.

More recently, EEG paradigms have also been used to investigate the connection between idiosyncratic fixation patterns and an individual's cortical responses. Using a fast‐periodic visual stimulation (FPVS) paradigm, Stacchi et al. (2019) found that locations that had longer viewing durations by an individual elicited stronger EEG responses than other facial positions at occipito‐temporal regions, suggesting that EM patterns could modulate neural responses in the face region. Though the FPVS paradigm has several strengths, it remains unclear in what time windows individuals' EM patterns could affect neural responses for faces. In the current study, we used a traditional temporal‐sensitive ERP approach to explore the questions. We particularly focused on the N170, a face‐sensitive ERP component, and, to a lesser extent, P1, as these components might be expected to vary in amplitude and latency based upon an individual's preferred fixation locations on a face. In the two experiments reported here, we aimed to examine the relationship between individual EM patterns and relevant face‐specific ERP components.

## 2 Experiment 1

In Experiment 1, following previous studies (e.g., Peterson and Eckstein 2012, 2013), we used a sequential face matching task, in which participants saw a series of target faces in a study block, and then in a later test block were required to distinguish the target faces from foils. We first conducted a free EM task in which participants observed faces with no restrictions; we analyzed these data to identify individuals' EM patterns for the faces. We expected that, consistent with previous studies, an upper‐focused EM pattern (focused on and around the eyes) and a lower‐focused EM pattern (focused on the nose and mouth) would be identified (e.g., Chuk, Chan, and Hsiao 2017). We then manipulated participants' fixation locations on the eye or on the nose to obtain their face identification performance and related ERPs. We wanted to determine whether the size of the N170 was due to visual information (with enhanced magnitude when the observer was focused on the facial eye‐region) or viewing preference (enhanced magnitude when the observer focused on their preferred facial location). Since upper‐focused individuals' preferred fixations were to the eye region, we expected that these observers would show a larger N170 when viewing the eyes than the nose no matter whether the effect was caused by their point of fixation (eyes) or their looking preference. For lower‐focused individuals, if viewing the eyes was the cause of the heightened N170 in previous studies, we would expect them to show a larger N170 on viewing the eyes than the nose, just like the upper‐focused group. If the larger N170 was caused by individuals' looking preference, these observers would show a larger N170 on viewing the nose than the eyes.

In addition, we wondered whether subsequent EMs would be modulated if the starting fixation positions were varied. Arizpe et al. (2012) found that the starting fixation location on faces affected later fixations. Specifically, they found that if the first fixation was around the eyes, the second fixation would be around the nose and mouth, while if the first fixation was around the nose and mouth, the second fixation would be around the eyes. In the current experiment, for the two‐fixation block, we also wondered whether the first fixation locations on the eyes or on the nose would be followed by significantly different second fixation locations.

### 2.1 Method

#### 2.1.1 Participants

Fifty (10 males) East Asian participants were recruited at The University of Hong Kong. Participants had normal or corrected‐to‐normal vision, age ranged from 18 to 28. Ethical approval was gained from the University of Hong Kong Human Research Ethics Committee.

#### 2.1.2 Materials

We used 48 grayscale Asian faces (24 males) with neutral expressions in this study, consisting of 16 faces (eight males) for each task block. We standardized the distances between the eyes and the mouth and cropped the faces into an elliptical shape. Participants were sitting 57 cm from the screen and faces subtended about 8° of visual angle horizontally and 11° vertically.

#### 2.1.3 Procedure

The experiment was a face identification task with three blocks: the free EM block, the two‐fixation block, and the one‐fixation block (Figure 1a). Generally, the difficulty of the task gradually increased. Participants were aware of the fixation number limitation at the beginning of each block, and they were able to take breaks between blocks and one or two breaks within blocks.

**FIGURE 1:**
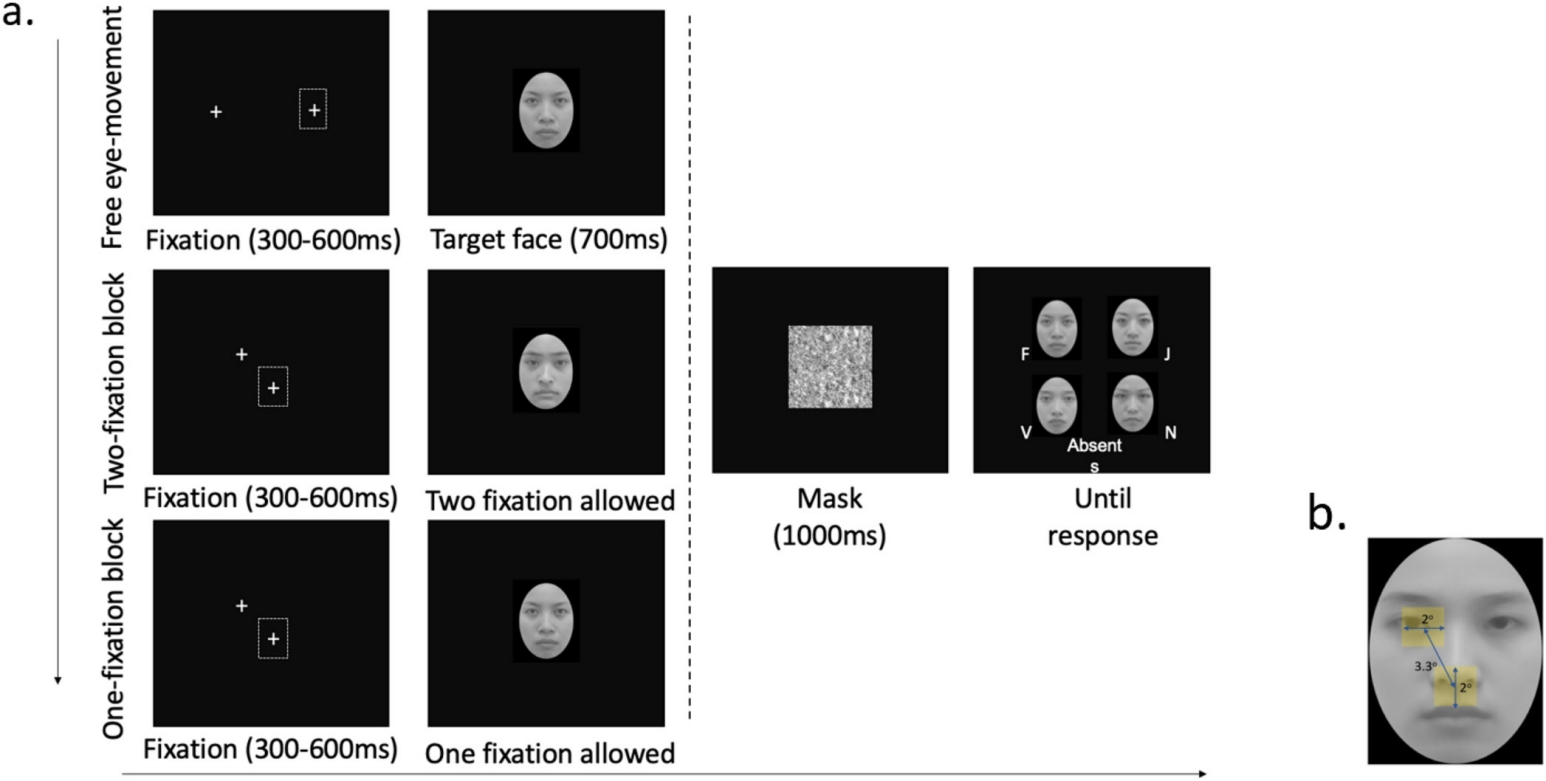
(a) Time course of a trial of the free EM condition (upper), two‐fixation condition (middle), and one‐fixation condition (lower). (b) Eye and nose fixation locations in the fixation number‐controlled blocks. The squares denote the acceptable areas of the fixation locations.

In the free EM block, each trial began with a fixation cross‐displayed on the left or right side of the screen (determined randomly). The cross was about 9° from the center of the screen, and participants were required to fixate within 1° of the cross for 300–600 ms until the cross disappeared. After that, a target face was displayed at the center of the screen for 700 ms, and then a mask covered the target face for 1000 ms. Following that, four faces were displayed, one at each quadrant, and participants indicated which one of these faces (or none of them) was the target face by pressing corresponding keys. There were 64 trials in total consisting of 32 trials in which the target face was “absent” and 32 trials in which the target face was present. Each quadrant had eight trials in which the target face appeared.

In the two‐fixation block, each trial began with a fixation cross randomly at the left eye (observers' view) corner or the bottom edge of the nose when the target face appeared (Figure 1b). The left eye inner corner was selected because the left bias has also been reported in some previous studies (e.g., Chuk, Crookes, et al. 2017; Hsiao and Cottrell 2008; Hsiao and Liu 2012; Or et al. 2015; Peterson and Eckstein 2012), particularly because the information at this location is especially diagnostic for face recognition (Royer et al. 2018). Participants were required to fixate within 1° of the cross for 300–600 ms, and then the cross disappeared, replaced by a target face shown at the center of the screen. In this block, participants were allowed to have two fixations on each target face. Once participants made a saccade from the second fixation location, a mask covered the target face for 1000 ms. After that, a response screen was displayed, as in the free EM block. There were 128 trials in total, with the fixation cross split equally between the left eye and the nose. Response accuracy was recorded in the same way as in the free EM block.

For the one‐fixation block, the procedure was similar to the two‐fixation block, except that a mask covered the target face after one fixation on the target face.

#### 2.1.4 Eye‐Tracking Recording and Data Analysis

Each participant's dominant eye was tracked using EyeLink 1000 plus (SR Research) at 1000 Hz sampling rate with default settings for cognitive research: saccade velocity threshold was 30°/s; saccade acceleration threshold was 8000°/square second; saccade motion threshold was 0.15°. A five‐point calibration was conducted at the beginning of each block.

The EMs were analyzed by the Eye Movement Hidden Markov Model toolbox (EMHMM, available at http://visal.cs.cityu.edu.hk/research/emhmm/). The EMHMM is a machine‐learning‐based approach for EM data analysis with the Hidden Markov Model (HMM). EMHMM assumes that a sequence of EMs can be modeled as a Markov process, with a series of hidden states (mapping to regions of interest [ROIs]) and transitions between states (mapped as saccades). Each participant's EMs during the free EM block were modeled using an HMM to represent the participant's EM pattern with reference to the participant's ROIs and the transitions among the ROIs. In this process, the ROIs are not pre‐set but generated from an individual's fixations, and the fixation density of an ROI follows a two‐dimensional Gaussian distribution. To determine the number of ROIs, we set a range of numbers of ROIs in advance, and the optimal number of ROIs is calculated using the variational Bayesian approach. The transition probabilities among the ROIs are summarized in a transition matrix. After that, all individuals' HMMs were clustered into subgroups according to their similarities using the Variational Bayesian Hierarchical Expectation Maximization (VHEM) algorithm (Lan et al. 2021). The algorithm required a range of numbers of subgroups, and it compared the separations of subgroups in those given numbers to determine the optimal number of subgroups for which the expected log‐likelihood of the model was the highest and produced a representative HMM for each subgroup. Each group‐representative HMM represents a general EM strategy discovered from the participants. Furthermore, to quantitatively assess the similarities between an individual's HMM and the group representative HMMs, we calculated the log‐likelihoods of the individual's EM data being generated by the group representative HMMs (for more details of EMHMM and its implementation, see Chuk et al. 2014).

In the current study, we used each participant's EMs to the target face in the free EM block to represent the personal preferred EM pattern. We set the range of ROIs from one to three, and we trained each participant's HMM with each number of ROIs 200 times. The HMM with the highest data likelihood was selected for the participant. We set the number of ROIs in the representative HMMs to two which was the median number of the ROIs among the individual models. Using the VBHEM algorithm (Lan et al. 2021) for clustering HMMs, we set the number of clusters from one to six, with the optimal number of representative patterns being determined as two. The log‐likelihoods are in Part 3 of Supporting Information.

#### 2.1.5 Electrophysiological Recording and Data Analysis

The EEG was recorded using an ANT EEGo system at 500 Hz sampling rate. We recorded signals with 64‐channel Ag/AgCl electrodes cap placed under the international 10–20 system, and all electrode impedances were kept below 20 kΩ during recording. The ground electrode was located between the Fpz and Fz electrodes.

EEG data were analyzed in MATLAB 2019a with EEGLAB toolbox (version 2019_1; Delorme and Makeig 2004) and ERPLAB toolbox (version 8.0; Lopez‐Calderon and Luck 2014). The EEG signals were filtered through a bandpass of 0.1–30 Hz (−6 dB/oct), and bad channels were detected and removed using EEGLAB's Clean_rawdata function with thresholds that flat line lasted for 5 s, maximum noise standard deviation was 8, and minimum correlation with nearby channels was 0.8. Independent component analysis (ICA) was performed (Jung et al. 2000), and EM artefacts were corrected by careful visual inspection referring to the ICLabel (Pion‐Tonachini et al. 2019). EEG signals were re‐referenced to the average after interpolation and the timestamps were moved 20 ms earlier because of a delayed time marker in the program. EEG epochs were extracted between −200 and 250 ms after the onset of the target face with baseline correction from −200 to 0 ms. Epochs with absolute voltage greater than 75 μV at any channels between −200 and 250 ms were removed. The P1 amplitude was measured as the grand peak between 70 and 150 ms post‐stimulus at Oz, where the P1 amplitude was elicited more strongly than at other electrodes. In the same vein, the N170 amplitude was measured as the grand peak between 150 and 220 ms post‐stimulus at P8, where the N70 amplitude was elicited more strongly than at other electrodes.^1^ The P1 and N170 latencies were measured as occurring at the peak amplitudes of the P1 and N170, respectively. In all analysis of ERPs, if a participant had fewer than 30 trials in one condition of a block, that participant would be excluded. In all analysis of behavioral and ERP ANOVAs, the Bonferroni correction was employed for post hoc comparisons to control Type I error.

For the free EM block, we demonstrated EMs and compared behavioral performance between groups. For fixation number‐controlled blocks, we first demonstrated EMs between groups and conducted 2 (initial fixation location: eye vs. nose, within participants) × 2 (EM pattern: upper‐focused vs. lower‐focused, between participants) mixed ANOVAs on behavioral performance to examine whether individual looking preference affects face identification performance modulated by initial fixation location. After that, to explore the related neural responses, we conducted 2 (initial fixation location: eye vs. nose) × 2 (EM pattern: upper‐focused vs. lower‐focused) mixed ANOVAs on P1 and N170 amplitudes and latencies respectively. For P1 latency comparisons between EM groups, we conducted conventional independent‐samples *t*‐tests.

### 2.2 Results

#### 2.2.1 EM Patterns in the Free EM Block and Behavioral Performance

As shown in Figure 2a, our clustering revealed an upper‐focused pattern with fixations mostly on the eyes, and a lower‐focused pattern with fixations around the nose. We further quantitatively assessed the similarities between an individual's pattern and the representative patterns by calculating the individual's log‐likelihoods of being generated by the representative pattern HMMs, and then assessed the individual's similarity along the upper‐focused pattern and the lower‐focused pattern dimension using the formula below:

**FIGURE 2:**
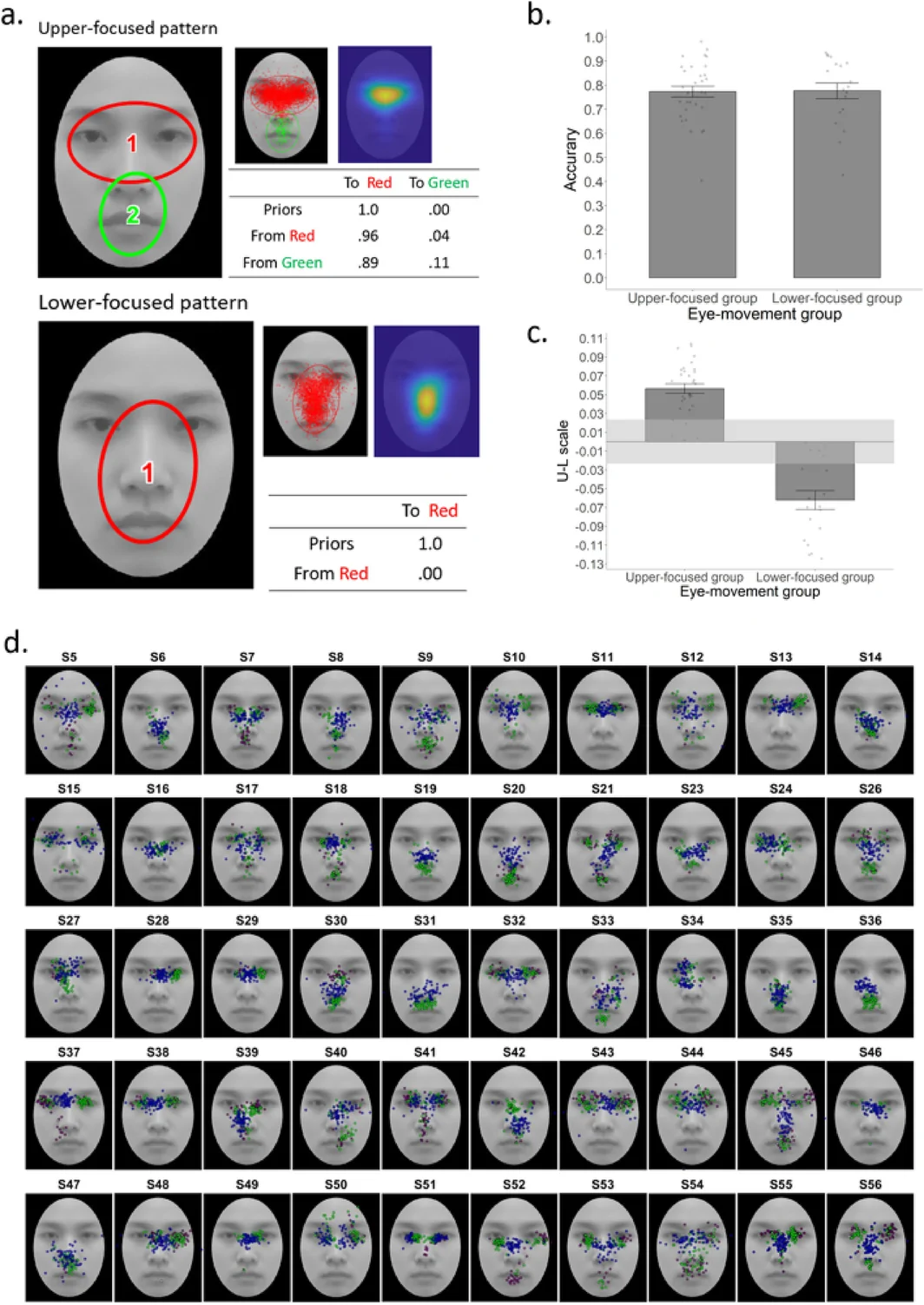
(a) The representative EM patterns generated from the free EM block. Here and elsewhere, the red and green ellipses are ROIs. The small images show the distribution of real fixations allocated to the ROIs. The tables show transition probabilities among the ROIs; priors denote the probabilities that the first fixation starts from the ROI. (b) Accuracy and (c) U‐L scale for the upper‐focused and lower‐focused groups in the free EM identification block. Error bars are SEs, dots are individual means. The gray transparent square indicates the near zero region using an MAD (Median Absolute Deviation) based threshold analysis. The results for the data excluding near zero participants are in Part 2 of Supporting Information. (d) Individuals' fixation locations on faces. The blue dots are the first fixations, the green dots are the second fixations, and the purple dots are the third fixations.

$\text{Upper}–\text{Lower Scale}=\left(U-L\right)/\left(|U|+|L|\right)$where *U* and *L* represent the individual's log‐likelihoods of being generated by the upper‐focused pattern HMM and the lower‐focused pattern HMM, respectively. A positive scale value means that an individual's pattern is more similar to the upper‐focused pattern, and vice versa for a negative scale value. We allocated participants with positive upper–lower scale (U‐L scale) values into the upper‐focused group, and participants with negative U‐L scale values into the lower‐focused group. In this way, we split participants into the upper‐focused group with 31 participants and the lower‐focused group with 19 participants, respectively (see Figure 2c). An independent *t*‐test was conducted on the U‐L scale for the EM pattern groups, *t* (48) = 11.82, *p* < 0.001, Cohen’s *d* = 3.44, suggesting that the EM patterns differ significantly between these two groups.

We compared the face identification performance between the upper‐focused and lower‐focused groups. An independent *t*‐test revealed no significant difference of accuracy between EM pattern groups, *t* (48) = 0.08, *p* = 0.94, Cohen’s *d* = 0.02, suggesting that identification performance did not differ between the two EM patterns significantly (see Figure 2b).

#### 2.2.2 EM Patterns and Behavioral Performance for the One‐Fixation Block and Two‐Fixation Blocks

2.2.2.1. One‐Fixation Block. Having previously clustered participants into representative EM pattern groups generated in the free EM block, we used a similar EMHMM analysis in the one‐fixation block to examine the effects of different fixation positions for each group (see Figure 3a). Note that the group assignment for each participant (upper‐focused or lower‐focused) was based on the free EM block, not the one‐fixation block.

**FIGURE 3:**
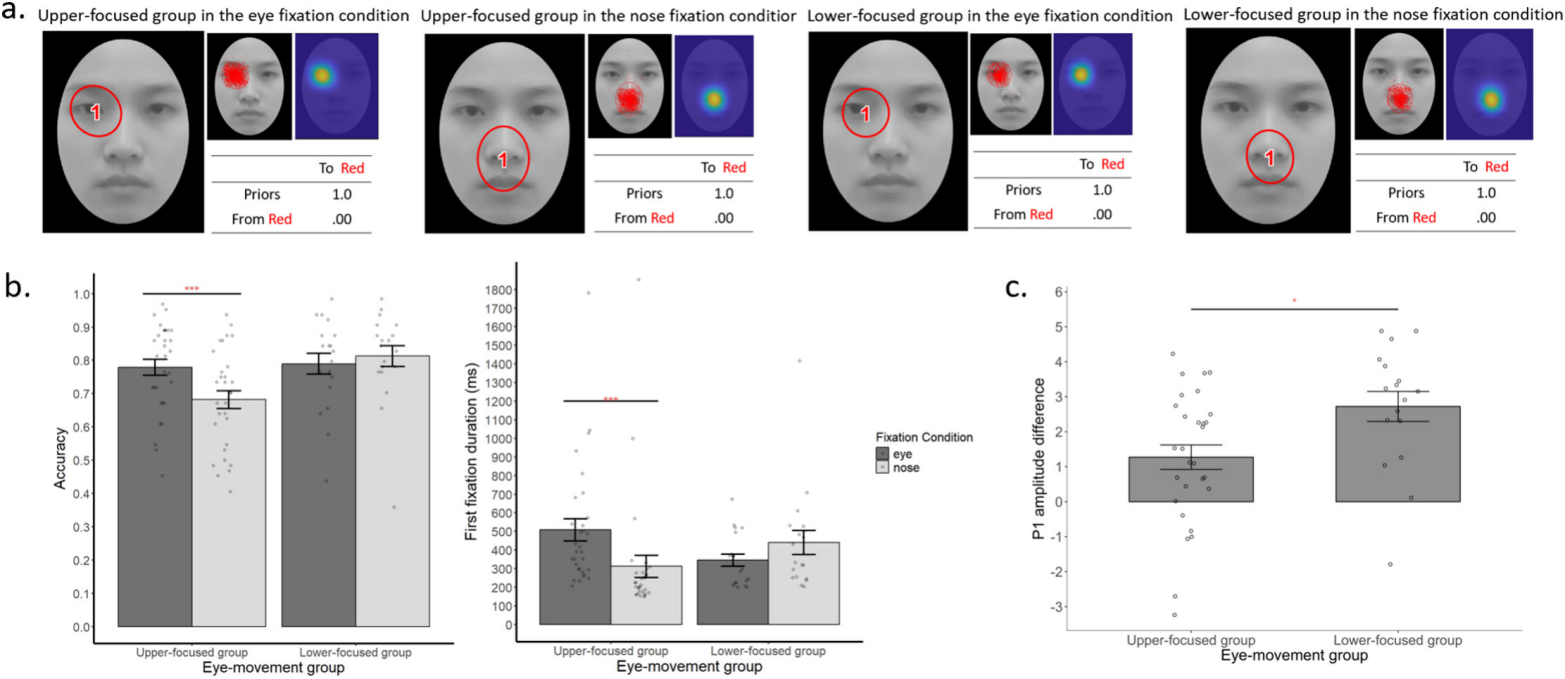
(a) The representative EM patterns generated from the one‐fixation block. Since participants only have one fixation in this block, there is no real transition among the ROIs. (b) Accuracy and the first fixation duration in the task. (c) The difference of P1 amplitudes between nose and eye fixation conditions. *, **, and *** indicate significant difference *p* < 0.05, *p* < 0.01, and *p* < 0.001, respectively.

A 2 (initial fixation location: eye vs. nose) × 2 (EM pattern: upper‐focused vs. lower‐focused) mixed ANOVA was conducted on accuracy (see Figure 3b). The main effect of initial fixation location was significant, F (1,48) = 8.55, *p* = 0.005, *η*_*p*_^*2*^ = 0.15. The main effect of EM pattern was marginally significant, F (1,48) = 3.32, *p* = 0.08, *η*_*p*_^*2*^ = 0.07. The interaction effect was significant, F (1,48) = 22.46, *p* < 0.001, *η*_*p*_^*2*^ = 0.32. Post hoc *t*‐tests of this interaction revealed that the upper‐focused group had higher identification accuracy for the eye condition than the nose condition, *t* (48) = 6.22, *p* < 0.001, while the lower‐focused group had comparable identification accuracy between the eye and the nose conditions, *t* (48) = −1.15, *p* > 0.05.

For fixation duration, neither of the main effects of initial fixation location or EM pattern were significant, F (1,48) = 1.72, *p* = 0.20, *η*_*p*_^*2*^ = 0.04 and F (1,48) = 0.05, *p* = 0.82, *η*_*p*_^*2*^ = 0.001, respectively. The interaction effect was significant, F (1,48) = 14.11, *p* < 0.001, *η*_*p*_^*2*^ = 0.23. Post hoc *t*‐tests of this interaction revealed that the upper‐focused group had longer fixation durations for the eye condition than the nose condition *t* (48) = 4.11, *p* < 0.001, while the lower‐focused group had comparable fixation durations between the eye and the nose conditions, *t* (48) = −1.56, *p* > 0.05. Since participants could only have one fixation on each target face, the first fixation duration was also the total duration.

2.2.2.2. Two‐Fixation Block. As with the one‐fixation block, participants were grouped based on the representative EM pattern groups generated in the free EM block, and then we ran the EMHMM analysis on data from the two‐fixation block for each group on each fixation condition (eye or nose) (see Figure 4a).

**FIGURE 4:**
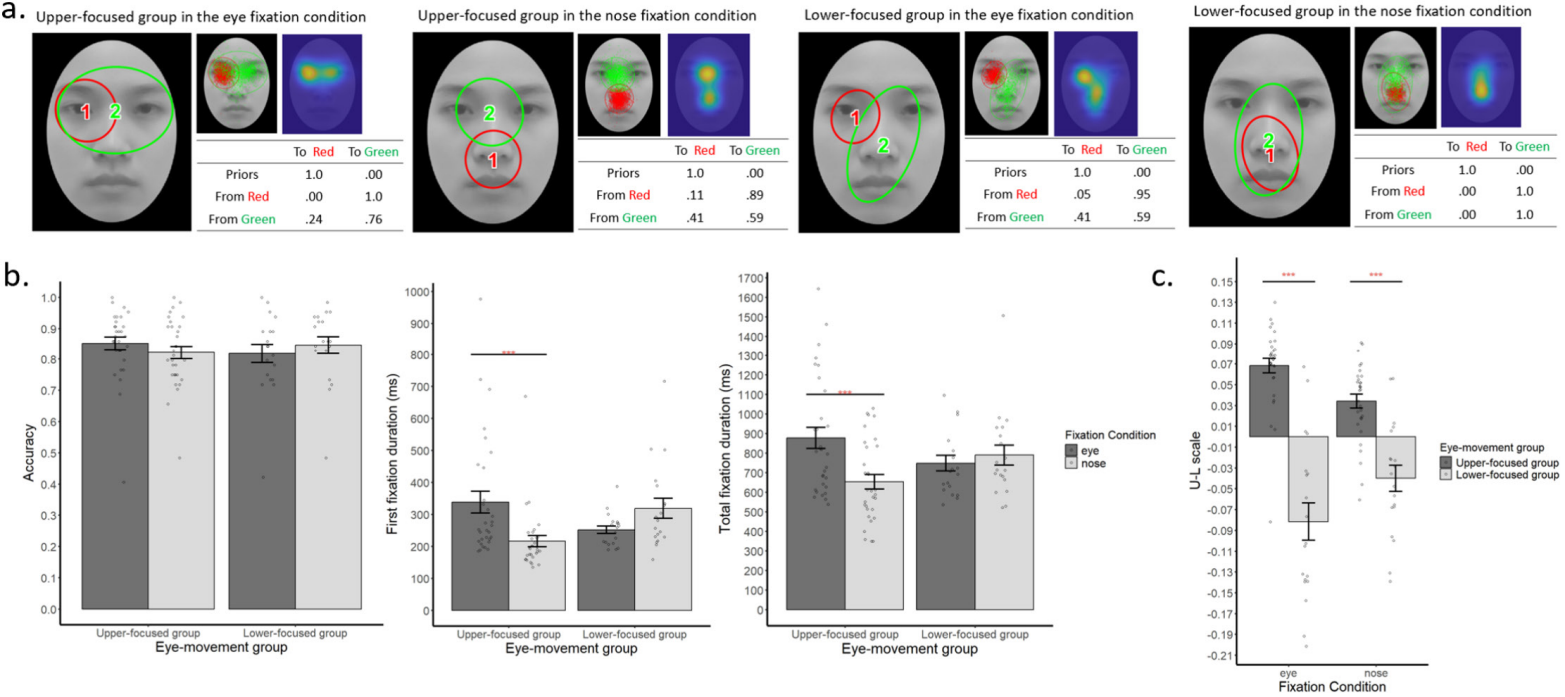
(a) Representative EM patterns generated from the two‐fixation block in the eye fixation condition and the nose fixation condition for the upper‐focused group and the lower‐focused group, respectively. (b) Accuracy, first fixation duration and total fixation durations in the two‐fixation block. (c) U‐L scale of the second fixation location of the two‐fixation block for the upper‐focused group and the lower‐focused group.

On each trial, the first fixation location was determined by the experiment (the required fixation position). To assess the eye movements to the second fixation location, we sorted the second fixations in the eye‐fixation and nose‐fixation conditions and calculated the similarity of each separately along the upper‐focused pattern and the lower‐focused pattern dimension using the U‐L scale formula as well. Therefore, each participant had separate U‐L scale values for the eye‐fixation condition and the nose‐fixation condition.

We conducted a 2 (initial fixation location: eye vs. nose) × 2 (EM pattern: upper‐focused vs. lower‐focused) mixed ANOVA on the U‐L scale value for the second fixation. The main effect of EM pattern was significant, F (1,48) = 72.5, *p* < 0.001, *η*_*p*_^*2*^ = 0.60. The interaction effect was significant, F (1,48) = 29.15, *p* < 0.001, *η*_*p*_^*2*^ = 0.38. Post hoc *t*‐tests revealed that U‐L scale value was bigger for the upper‐focused group than the lower‐focused group for both the eye‐fixation condition (*t* (48) = 9.01, *p* < 0.001) and the nose‐fixation condition (*t* (48) = −5.73, *p* < 0.001), suggesting that the upper‐focused group showed more upper‐focused patterns than the lower‐focused group in both eye‐fixation and nose‐fixation conditions for the second fixation location (see Figure 4c).

For behavioral performance (see Figure 4b), a 2 (initial fixation location: eye vs. nose) × 2 (EM pattern: upper‐focused vs. lower‐focused) mixed ANOVA was performed on accuracy. The main effects of initial fixation location and EM pattern were not significant, F (1,48) = 0.03, *p* = 0.87, *η*_*p*_^*2*^ = 0.001 and F (1,48) = 0.02, *p* = 0.89, *η*_*p*_^*2*^ = 0.000 for fixation location and EM pattern, respectively. The interaction between initial fixation location and EM pattern was significant, F (1,48) = 9.96, *p* = 0.003, *η*_*p*_^*2*^ = 0.17. Post hoc *t*‐tests revealed that the upper‐focused group had marginally significant better performance on the eye condition than the nose condition, *t* (48) = 2.70, *p* = 0.06, while the lower‐focused group had comparable performance on the eye and the nose conditions, *t* (48) = −1.90, *p* > 0.05.

For the first fixation duration, the main effects of initial fixation location and EM pattern were not significant, F (1,48) = 1.74, *p* = 0.20, *η*_*p*_^*2*^ = 0.04 and F (1,48) = 0.06, *p* = 0.81, *η*_*p*_^*2*^ = 0.001, respectively. The interaction effect was significant, F (1,48) = 20.54, *p* < 0.001, *η*_*p*_^*2*^ = 0.30. Post hoc analysis of this interaction revealed that participants in the upper‐focused group had longer fixation durations for the eye condition than the nose condition *t* (48) = 4.75, *p* < 0.001, while those in the lower‐focused group had comparable fixation durations between the eye and the nose conditions, *t* (48) = −2.04, *p* > 0.05.

For the total fixation duration, the main effect of initial fixation location was significant, F (1,48) = 7.31, *p* = 0.01, *η*_*p*_^*2*^ = 0.13. The main effect of EM pattern was not significant, F (1,48) = 0.003, *p* = 0.96, *η*_*p*_^*2*^ = 0.000. The interaction effect was significant, F (1,48) = 15.31, *p* < 0.001, *η*_*p*_^*2*^ = 0.24. Post hoc analysis of this interaction revealed that the upper‐focused group had longer fixation duration for the eye condition than the nose condition *t* (48) = 5.37, *p* < 0.001, while the lower‐focused group had comparable duration between the eye and the nose conditions, *t* (48) = −0.77, *p* > 0.05.

To compare the behavioral performance in the one‐fixation and two‐fixation blocks, a 2 (EM pattern: upper‐focused vs. lower‐focused) × 2 (initial fixation location: eye vs. nose) × 2 (Fixation number block: one‐fixation vs. two‐fixation) three‐way mixed ANOVA was performed on accuracy. The three‐way interaction effect was significant, F (1,48) = 5.38, *p* = 0.025, *η*_*p*_^*2*^ = 0.10. We then separated the data for the upper‐focused group and the lower‐focused group; the results showed that the interaction between initial fixation location and fixation number block was significant for the upper‐focused group, F (1,30) = 16.4, *p* < 0.001, *η*_*p*_^*2*^ = 0.35, but not for the lower‐focused group, F (1,18) = 0.04, *p* = 0.85, *η*_*p*_^*2*^ = 0.002, suggesting that fixation number modulated identification performance for the upper‐focused group but not for the lower‐focused group.

#### 2.2.3 ERP Components

2.2.3.1. One‐Fixation Block. Four participants (three from the upper‐focused group and one from the lower‐focused group) were excluded in this block because they had fewer than 30 trials in at least one fixation condition. Therefore, there were 46 participants in total, specifically 29 participants in the upper‐focused group and 17 participants in the lower‐focused group, who remained for ERP analysis. The average trial numbers for analysis were 61.5 and 62.0 trials in the eye‐fixation and nose‐fixation conditions, respectively.

For P1 amplitude, a 2 (initial fixation location: eye vs. nose) × 2 (EM pattern: upper‐focused vs. lower‐focused) mixed ANOVA was conducted. The main effect of EM pattern was not significant, F (1,44) = 1.42, *p* = 0.24, *η*_*p*_^*2*^ = 0.03. The main effect of initial fixation location was significant, F (1,44) = 48.00, *p* < 0.001, *η*_*p*_^*2*^ = 0.52. The interaction effect was significant, F (1,44) = 15.80, *p* = 0.011, *η*_*p*_^*2*^ = 0.14. Post hoc *t*‐tests revealed that P1 amplitude was larger for the nose fixation condition than the eye fixation condition for both the upper‐focused group (*t* (44) = 3.53, *p* = 0.006) and the lower‐focused group (*t* (44) = 6.02, *p* < 0.001), suggesting that fixating on the nose elicited larger P1 amplitude than on the eyes for both EM groups (see Figure 5).

**FIGURE 5:**
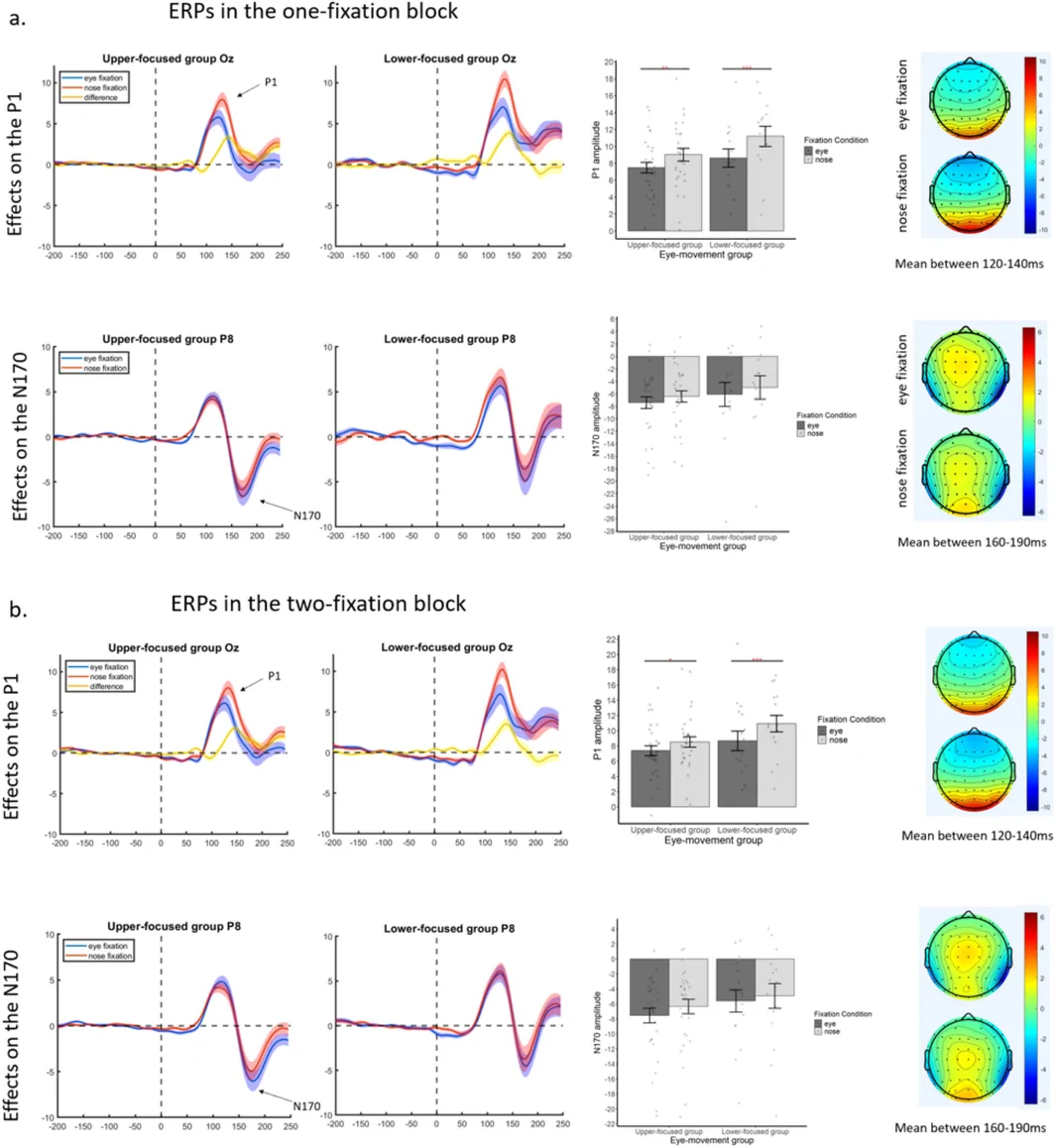
(a) and (b) Waveforms, bar graphs and topographic maps at Oz for the P1 and at P8 for the N170 in the one‐fixation block and two‐fixation block, respectively.

To further explore the interaction between fixation location and EM pattern for P1, we first calculated the difference of P1 amplitudes between the eye and the nose fixation conditions and then conducted an independent‐samples *t*‐test on the P1 difference for the upper‐focused and the lower‐focused groups (see Figure 3c). It revealed that the upper‐focused group had smaller P1 amplitude difference than the lower‐focused group (mean: 1.38 vs. 3.11, *t* (44) = −2.64, *p* = 0.011, Cohen’s *d* = 0.81).

For P1 latency, the main effect of initial fixation location was significant, F (1,44) = 15.53, *p* < 0.001, *η*_*p*_^*2*^ = 0.26, suggesting that P1 latency was longer for the nose condition than the eye condition. No other significant effects were found for P1 latency (*p*_s_ > 0.05).

For N170 amplitude, the main effect of initial fixation location was significant, F (1,44) = 13.88, *p* < 0.001, *η*_*p*_^*2*^ = 0.24. No other significant effects were found (*p*_s_ > 0.05).

For N170 latency, the interaction effect was significant, F (1,44) = 6.28, *p* = 0.02, *η*_*p*_^*2*^ = 0.16. Post hoc *t*‐tests revealed that N170 latency was longer for the nose fixation condition than the eye fixation condition for the upper‐focused group (*t* (44) = 2.85, *p* = 0.006) but not for the lower‐focused group (*t* (44) = 0.98, *p* > 0.05). No other significant effects were found (*p*_s_ > 0.05).

Given that P1 peaks were modulated by fixation location and EM group at P8 electrode pair (the interaction between initial fixation location and EM pattern was significant, F (1,44) = 6.39, *p* = 0.02, *η*_*p*_^*2*^ = 0.13), we conducted peak‐to‐peak analysis on P1‐to‐N170 amplitude to investigate whether the differences of N170 peaks arose from the different P1 baselines. The amplitude was calculated by P1 peak voltage minus N170 peak voltage (see Figure 6a).

**FIGURE 6:**
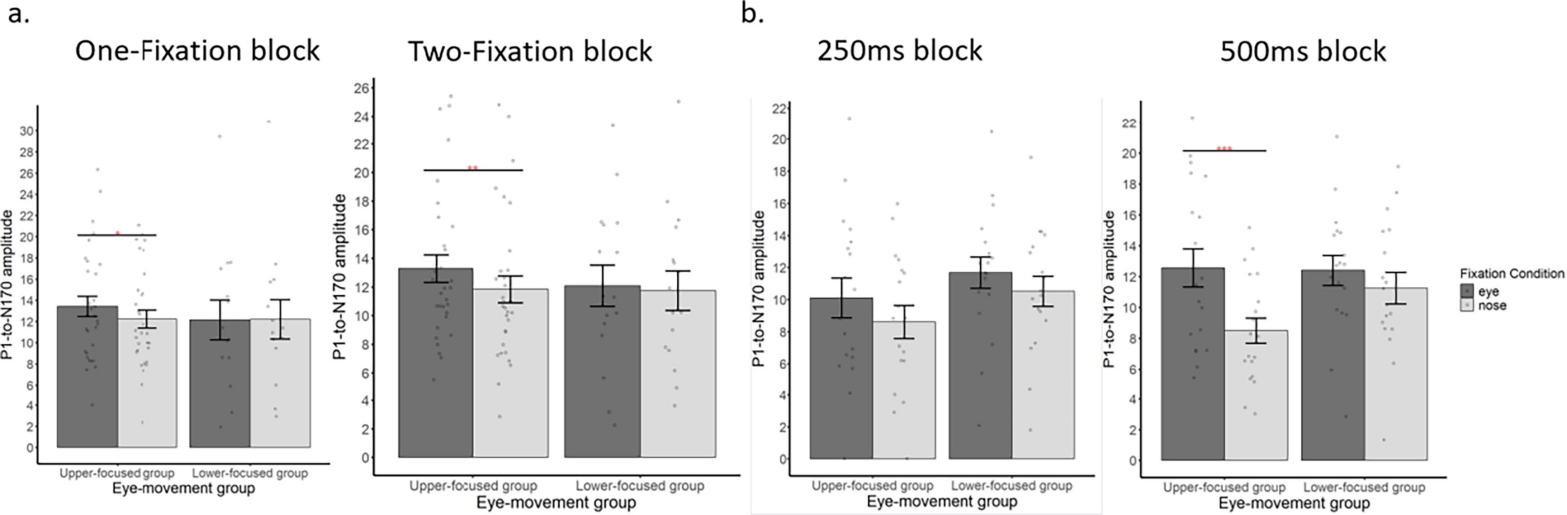
(a) The P1‐to‐N170 amplitudes at P8 in bar graphs in Experiment 1. (b) The P1‐to‐N170 amplitudes at P8 in bar graphs in Experiment 2.

The main effect of initial fixation location was marginally significant, F (1,44) = 2.85, *p* = 0.098, *η*_*p*_^*2*^ = 0.06. The interaction between fixation location and EM pattern was significant, F (1,44) = 4.11, *p* = 0.049, *η*_*p*_^*2*^ = 0.09. Post hoc *t*‐tests revealed that the amplitude was larger in the eye fixation condition than in the nose fixation condition for the upper‐focused group (*t* (44) = 3.06, *p* = 0.023), but no significant difference was found between fixation conditions for the lower‐focused group, *t* (44) = 0.21, *p* > 0.05. No other significant effects were found (*p*_s_ > 0.05).

2.2.3.2. Two‐Fixation Block. One participant from the upper‐focused group was excluded because of having fewer than 30 trials in at least one fixation condition. Therefore, 49 participants remained for the ERP analysis: 31 and 19 participants from the upper‐focused group and the lower‐focused group, respectively. The average trial numbers remaining for analysis were 59.8 and 60.6 trials in the eye‐fixation and nose‐fixation conditions respectively in this block.

For P1 amplitude, the main effect of initial fixation location was significant, F (1,47) = 38.30, *p* < 0.001, *η*_*p*_^*2*^ = 0.45. The interaction effect was significant, F (1,47) = 4.46, *p* = 0.040, *η*_*p*_^*2*^ = 0.09. Post hoc *t*‐tests revealed that P1 amplitude was larger for the nose fixation condition than the eye fixation condition for both the upper‐focused group (*t* (47) = 3.27, *p* = 0.012) and the lower‐focused group (*t* (47) = 5.31, *p* < 0.001), suggesting that fixating on the nose elicited larger P1 amplitude than on the eyes for both EM groups. The independent‐samples *t*‐test on the P1 difference between fixation conditions for the upper‐focused and the lower‐focused groups revealed that the upper‐focused group had smaller P1 amplitude difference than the lower‐focused group (Mean: 1.31 vs. 2.82, *t* (47) = −2.11, *p* = 0.004, Cohen’s *d* = 0.62. See Figure 5).

For P1 latency, the main effect of initial fixation location was significant, F (1,47) = 20.96, *p* < 0.001, *η*_*p*_^*2*^ = 0.31. No other significant effects were found (*p*_s_ > 0.05).

For N170 amplitude, the main effect of initial fixation location was significant, F (1,47) = 17.45, *p* < 0.001, *η*_*p*_^*2*^ = 0.27. No other significant effects were found (*p*_s_ > 0.05). For N170 latency, the interaction effect was marginally significant, F (1,47) = 3.66, *p* = 0.062, *η*_*p*_^*2*^ = 0.07. No other significant effects were found (*p*_s_ > 0.05).

### 2.3 Discussion

In Experiment 1, we identified two EM patterns for viewing faces, and we found differences in both fixation effects and ERP components between the groups. Specifically, the lower‐focused group had comparable identification performance and fixation durations between the eye‐fixation and the nose‐fixation conditions in both one‐fixation and two‐fixation blocks, and showed no differences in P1‐N170 amplitude. In contrast, upper‐focused individuals had better identification performance along with longer fixation durations in the eye‐fixation condition than in the nose‐fixation condition in the one‐fixation block, and they showed greater P1‐N170 peak‐to‐peak amplitude for preferred than non‐preferred fixation conditions. The result for the upper‐focused group supports earlier findings that looking preference optimizes identification performance (Peterson and Eckstein 2013). However, in the two‐fixation block, the upper‐focused group also had longer fixation durations in the eye‐fixation condition than in the nose‐fixation condition, but they performed comparably in these two fixation conditions. Since the total duration of the two‐fixation block was longer than that of the one‐fixation block, and simply longer presentation time of study faces alone could improve identification performance (Arizpe et al. 2019), it is possible that longer fixation duration on the non‐preferred location (such as on the nose for the upper‐focused group) improved identification performance as well as fixating on the preferred location. However, despite the forced initial fixation locations, the second fixations for the upper‐focused group reverted to their preferred pattern. So, it is also possible that the second fixation contributes to improving their identification performance in the nose‐initiated fixation condition because the second fixation is on their preferred location. To distinguish these two possibilities, we conducted Experiment 2 in which participants would have a single fixation on faces for different durations to examine whether the performance difference for the upper‐focused group was caused by potential differences in fixation duration. We conducted a face identification task with three blocks: a free EM block in which participants observed faces with no restrictions, and two guided fixation blocks in which participants were guided to fixate either on the eye or on the nose of a target face for 250 or 500 ms. If longer duration was not the factor for the elimination of the significant difference between fixation locations for the upper‐focused group, we expected that the upper‐focused group would perform better when fixation was on the eye than on the nose in both guided fixation blocks.

## 3 Experiment 2

### 3.1 Method

#### 3.1.1 Participants and Materials

To overcome the unbalanced sample sizes between the upper‐focused and lower‐focused groups in the previous experiment, in Experiment 2, we recruited equal numbers of participants for each group. We used the group representative EM patterns generated in Experiment 1 as templates, and we compared each participant's eye movements in the free EM block in Experiment 2 to these templates, allocating the participant to the group that best matched their performance. Estimated by a power analysis with effect size f = 0.25, α = 0.05, power = 0.90, using G*Power (Faul et al. 2007), the total sample size was 46 with 23 participants in each group. Therefore, a total of 46 East Asian participants (13 males) were recruited at The University of Hong Kong. Once one group reached 23 participants, additional participants were only included in the experiment if they displayed the other EM pattern; those showing the fixation pattern for the group that was full were discarded from the experiment and not further tested. Testing concluded when both groups had reached a size of 23 participants.^2^ Eventually, we had 24 participants in the upper‐focused group and 22 participants in the lower‐focused group. Images were the same as in Experiment 1.

#### 3.1.2 Procedure

All participants completed the free EM block followed by the guided fixation blocks. The procedure of the guided fixation blocks was similar to the one‐fixation block in Experiment 1, except that the presentation duration of target faces was fixed at 250 or 500 ms, and participants were asked to maintain fixation on the location that was cued by the cross until the target face disappeared (Figure 7). There were 160 trials in each block, including 80 trials in each fixation location, and the order of the guided fixation blocks was counterbalanced among participants.

**FIGURE 7:**
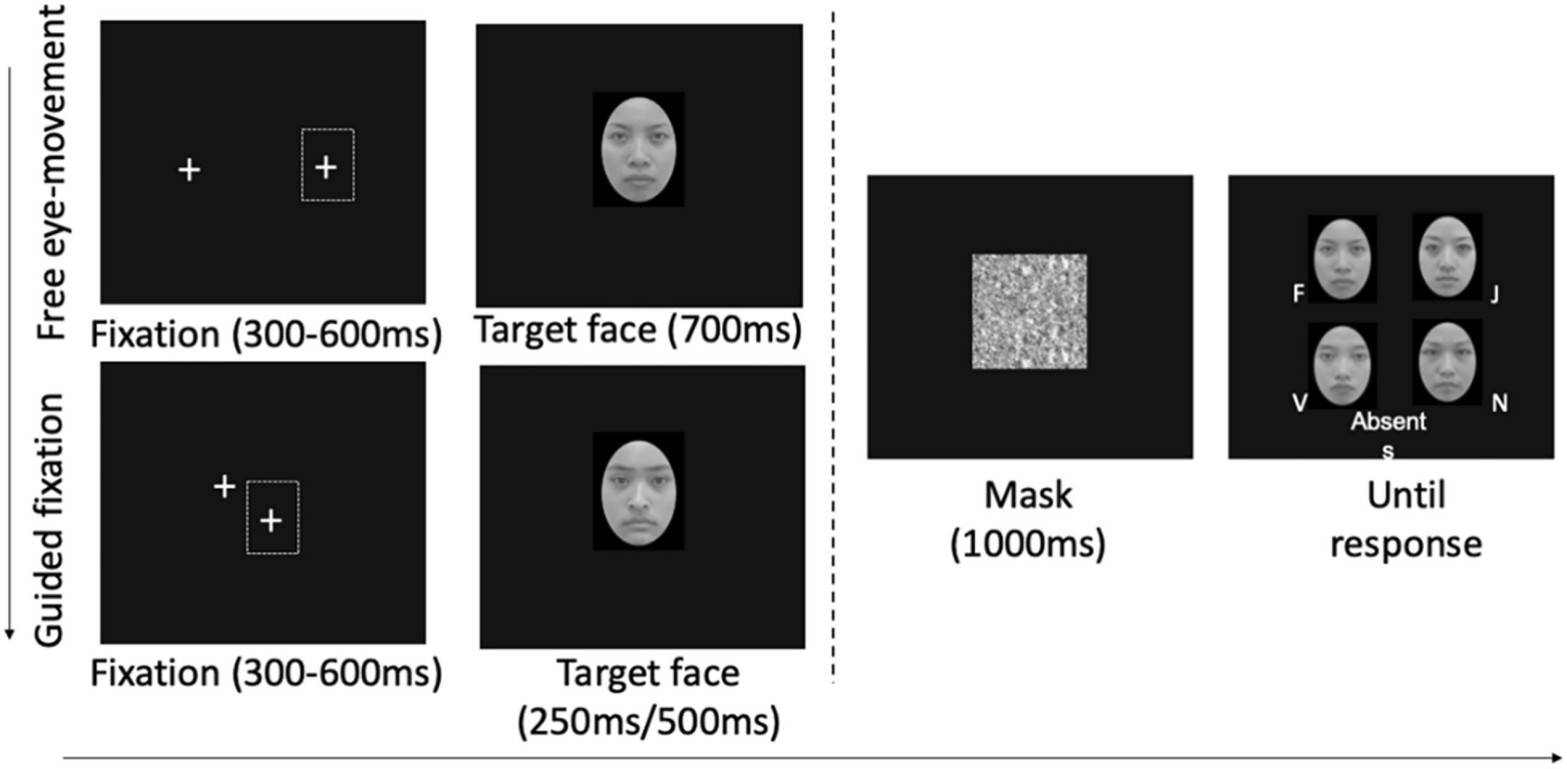
The time course of trials for the free EM block (upper) and the guided fixation blocks (lower) of 250 ms and 500 ms durations, respectively.

### 3.2 Results

#### 3.2.1 Behavioral Performance

For identification performance in the free EM block, consistent with Experiment 1, an independent *t*‐test revealed no significant difference of accuracy between EM pattern groups, *t* (44) = 1.07, *p* = 0.29, Cohen’s *d* = 0.32 (see Figure 8).

**FIGURE 8:**
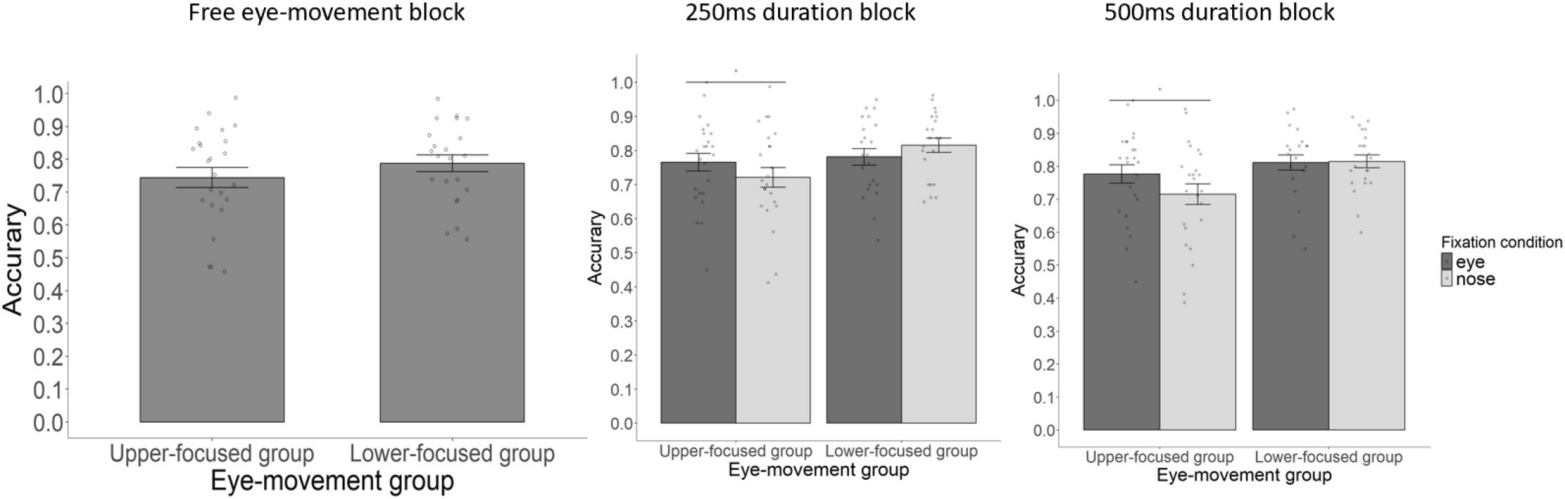
Accuracy in the free eye‐movement block and 250 and 500 ms duration blocks.

In the 250 ms duration block, a 2 (Initial fixation location: eye vs. nose) × 2 (EM pattern: upper‐focused vs. lower‐focused) mixed ANOVA was conducted on accuracy. The main effects of initial fixation location and EM pattern were not significant, F (1,44) = 0.26, *p* = 0.62, *η*_*p*_^*2*^ = 0.006, and F (1,44) = 2.58, *p* = 0.12, *η*_*p*_^*2*^ = 0.06, respectively. The interaction between fixation location and EM pattern was significant, F (1,44) = 13.95, *p* < 0.001, *η*_*p*_^*2*^ = 0.24. Post hoc *t*‐tests of this interaction revealed that the upper‐focused group had higher identification accuracy for the eye condition than the nose condition, *t* (44) = 3.07, *p* = 0.02, while the lower‐focused group did not show differences between the eye and the nose conditions, *t* (44) = −2.23, *p* > 0.05.

In the 500 ms duration block, the main effect of initial fixation location was significant, F (1,44) = 4.73, *p* = 0.035, *η*_*p*_^*2*^ = 0.10. The main effect of EM pattern was marginally significant, F (1,44) = 3.72, *p* = 0.060, *η*_*p*_^*2*^ = 0.08. The interaction between fixation location and EM pattern was significant, F (1,44) = 4.55, *p* = 0.039, *η*_*p*_^*2*^ = 0.10. Post hoc *t*‐tests of this interaction revealed that the upper‐focused group had higher identification accuracy for the eye condition than for the nose condition, *t* (44) = 3.30, *p* = 0.01, while the lower‐focused group did not show differences between the eye and the nose conditions, *t* (44) = 0.15, *p* > 0.05.

To compare the behavioral performance between 250 and 500 ms blocks, a 2 (initial fixation location: eye vs. nose) × 2 (fixation duration: 250 ms vs. 500 ms) × 2 (EM pattern: upper‐focused vs. lower‐focused) 3‐way mixed ANOVA was conducted on accuracy. Only the interaction effect between fixation location and EM pattern was significant, F (1,44) = 14.03, *p* < 0.001, *η*_*p*_^*2*^ = 0.24. No other effects were significant (*p*_s_ > 0.05), suggesting that fixation duration did not modulate identification performance in this experiment.

#### 3.2.2 ERP Components in the 500 ms Fixation Duration Block

Nine participants were excluded for ERP analysis in this block because of high data noise. Therefore, 37 participants (19 in the upper‐focused group and 18 in the lower‐focused group) remained. Results are shown in Figure 9.

**FIGURE 9:**
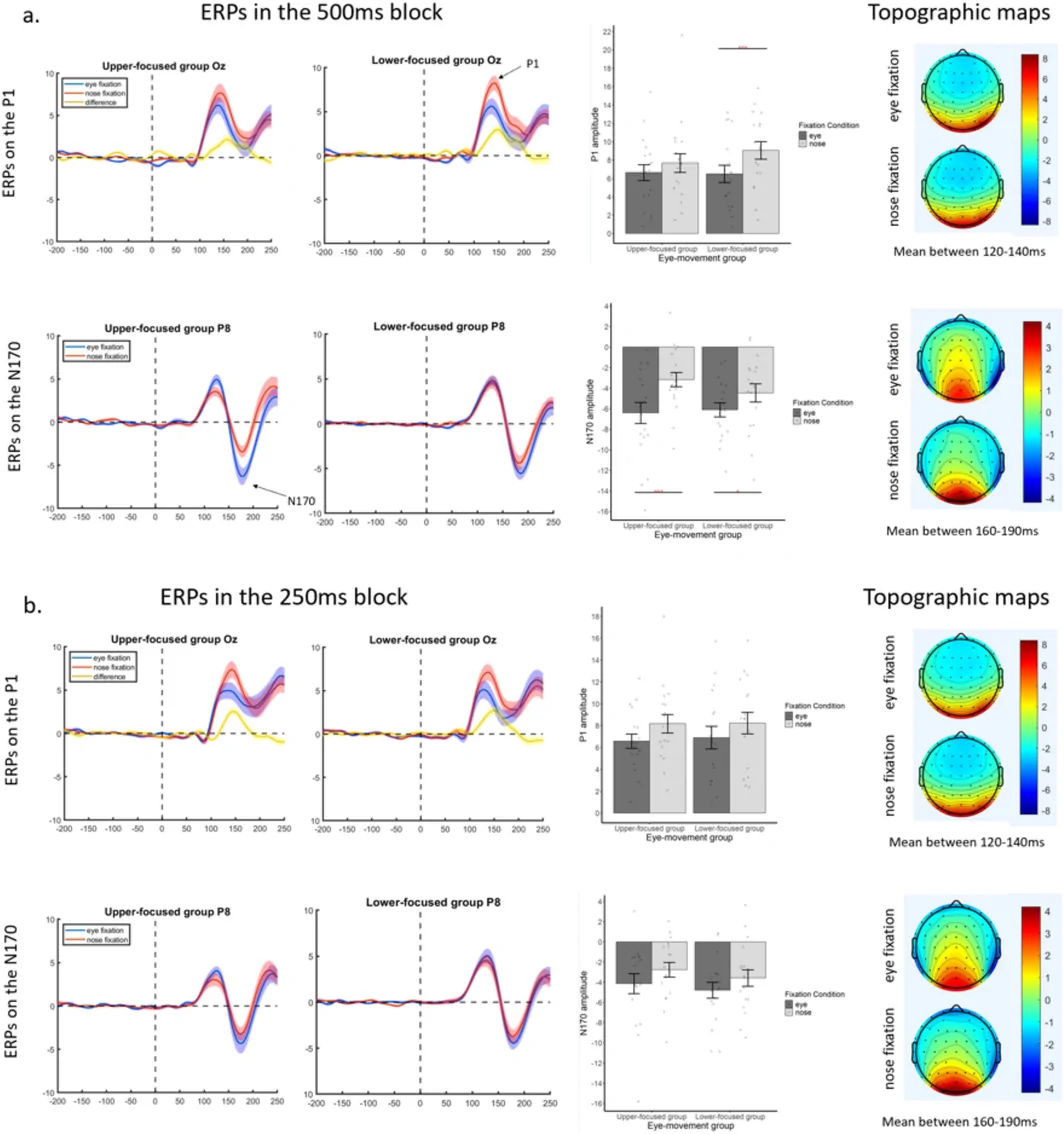
(a) and (b) Waveforms, bar graphs, and topographic maps at Oz for the P1 and at P8 for the N170 in the 500 and 250 ms durations block, respectively.

For P1 amplitude, a 2 (initial fixation location: eye vs. nose) × 2 (EM pattern: upper‐focused vs. lower‐focused) mixed ANOVA was conducted. The main effect of initial fixation location was significant, F (1,35) = 33.74, *p* < 0.001, *η*_*p*_^*2*^ = 0.49, suggesting that P1 amplitude was larger for the nose condition than for the eye condition. The interaction effect was significant, F (1,35) = 6.03, *p* = 0.019, *η*_*p*_^*2*^ = 0.15. Post hoc *t*‐tests revealed that P1 amplitude was larger for the nose fixation condition than the eye fixation condition for the lower‐focused group (*t* (35) = 5.77, *p* < 0.001) but showed no differences between fixation conditions for the upper‐focused group (*t* (35) = 2.40, *p* > 0.05). No other effects were significant (*p*_s_ > 0.05).

For P1 latency, the main effect of initial fixation location was significant, F (1,35) = 10.33, *p* = 0.003, *η*_*p*_^*2*^ = 0.23, suggesting that P1 latency was longer for the nose condition than the eye condition. No other significant effects were found for P1 latency (*p*_s_ > 0.05).

For N170 amplitude, the main effect of initial fixation location was significant, F (1,35) = 42.37, *p* < 0.001, *η*_*p*_^*2*^ = 0.55. The interaction between fixation location and EM pattern was significant, F (1,35) = 4.51, *p* = 0.041, *η*_*p*_^*2*^ = 0.11. Post hoc *t*‐tests revealed that the N170 was larger in the eye fixation condition than in the nose fixation condition for the upper‐focused group (*t* (35) = 6.12, *p* < 0.001) and the lower‐focused group, *t* (35) = 3.06, *p* = 0.03. No other significant effects were found (*p*_s_ > 0.05). No significant effects were found for N170 latency (*p*_s_ > 0.05).

Given that P1 peaks were modulated by fixation location and EM group at the P8 electrode pair (the interaction between initial fixation location and EM pattern was significant, F (1,35) = 6.45, *p* = 0.016, *η*_*p*_^*2*^ = 0.16), we conducted peak‐to‐peak analysis on P1‐to‐N170 amplitude to investigate whether the differences of N170 peaks arose from the different P1 baselines (see Figure 6b).

The main effect of initial fixation location was significant, F (1,35) = 31.77, *p* < 0.001, *η*_*p*_^*2*^ = 0.48. The interaction between fixation location and EM pattern was significant, F (1,35) = 9.93, *p* = 0.003, *η*_*p*_^*2*^ = 0.22. Post hoc *t*‐tests revealed that the amplitude was larger in the eye fixation condition than in the nose fixation condition for the upper‐focused group (*t* (35) = 6.30, *p* < 0.001), but no significant difference was found between fixation conditions for the lower‐focused group, *t* (35) = 1.73, *p* > 0.05. No other significant effects were found (*p*_s_ > 0.05).

#### 3.2.3 ERP Components in the 250 ms Fixation Duration Block

Ten participants were excluded from ERP analysis in this block because of high data noise. Therefore, 36 participants (18 in each group) remained. Results are shown in Figure 9.

For P1 amplitude, the main effect of initial fixation location was significant, F (1,34) = 28.89, *p* < 0.001, *η*_*p*_^*2*^ = 0.46. No other differences were significant (*p*_s_ > 0.05). For P1 latency, the main effect of initial fixation location was significant, F (1,34) = 14.23, *p* < 0.001, *η*_*p*_^*2*^ = 0.30. No other significant effects were found for P1 latency (*p*_s_ > 0.05).

For N170 amplitude, the main effect of initial fixation location was significant, F (1,34) = 18.23, *p* < 0.001, *η*_*p*_^*2*^ = 0.35. No other significant effects were found for N170 amplitude (*p*_s_ > 0.05). For N170 latency, no significant effects were found (*p*_s_ > 0.05).

### 3.3 Discussion

In Experiment 2, after equating the sample size of the EM groups and limiting the fixation number and the presentation duration of the study faces, we found that the upper‐focused group performed better for the eye‐fixation condition than the nose‐fixation condition in both 250 and 500 ms blocks, which are consistent with the result in the one‐fixation block in Experiment 1, and we replicated the larger P1‐N170 peak‐to‐peak amplitude for upper‐focused but not lower‐focused observers when fixating on the eyes. In addition, no significant differences were found between the 250 and 500 ms blocks, suggesting that simply prolonging fixation duration did not affect identification performance between fixation locations. For the lower‐focused group, we also replicated the findings in Experiment 1, in that they performed equally well between those two fixation conditions. Taken overall, these results show that the pattern of results in Experiment 1 was not affected by different sample sizes in the two groups of participants or differences in the duration of stimulus visibility.

## 4 General Discussion

In the current study, we explored the relationship between individual differences in EM patterns, face identification performance, and the electrophysiological brain response. Using the EMHMM approach (Chuk et al. 2014), we modelled EMs for each observer when they viewed faces and then clustered them into two distinct EM patterns. No significant overall difference in face identification performance was found between individuals using these two different EM patterns. However, the upper‐focused group performed better when they were asked to fixate on the eyes of faces than on the nose, whereas the lower‐focused group showed no significant differences in identification performance between fixations on the eyes and the nose. For neural responses, we found that the N170 amplitude was larger when fixations were on the eye region than on the nose region for both groups. However, after taking the P1 baseline into consideration, we found that the upper‐focused group showed a larger P1‐to‐N170 peak‐to‐peak amplitude when they were asked to fixate on the eyes than on the nose. The peak‐to‐peak amplitudes remained larger in the eye‐fixation condition than the nose‐fixation condition after equating fixation durations. In contrast, the lower‐focused group showed no significant differences in identification performance, fixation duration, or P1‐to‐N170 peak‐to‐peak amplitude between fixations on the eyes and the nose. These findings suggest that for upper‐focused observers, information from upper halves of faces is prioritized compared with that from lower halves: the face‐sensitive electrophysiological signal amplitude is larger and face identification performance is better for these observers when fixating on the upper regions of a face. In contrast, looking at upper or lower halves does not affect face identification performance or N170 amplitude for lower‐focused observers.

In fixation‐controlled blocks in Experiment 1, if the initial fixations were on the eyes, upper‐focused individuals had better performance and longer fixation durations compared with fixations on the nose. If the initial fixation was on the lower half of a face, these participants made a saccade to the upper half of the face quickly. Even though they knew that the target face would disappear if they made a saccade, they did not fixate on the nose for as long as on the eye. The better performance in the eye‐fixation condition was also found in Experiment 2 in which the fixation durations were held equal between fixation conditions. Moreover, no significant differences were found between 250 and 500 ms durations for the upper‐focused group, indicating that 250 ms fixation duration is sufficient for identifying faces, and longer fixation duration did not improve performance or diminish the difference between fixation locations. These results are inconsistent with the study that found that face identification performance could increase by prolonging presentation time of study faces (Arizpe et al. 2019). Instead, our results suggest that participants can reach maximal identification performance when given one fixation with a short duration (250 ms in the experiment), and their performance stays at that level even if the fixation duration is extended (500 ms in the experiment; see also Hsiao and Cottrell 2008). Looking at an upper‐focused individual's preferred location appears to be an effective strategy for optimizing face identification performance.

In contrast, lower‐focused individuals showed similar identification performance and fixation durations between the eye and the nose fixation conditions. These results are partially consistent with Stacchi et al.'s (2019) study, in which they found that individuals had longer fixation durations on preferred facial positions, and looking at an individual's preferred location elicited stronger face‐sensitive neural responses than other facial positions. It is also partially consistent with previous studies that have found that idiosyncratic EM patterns serve to optimize identification performance (Peterson and Eckstein 2012, 2013). Note that the fixation locations where participants were looking differ among these studies. In the study of Stacchi et al. (2019), participants were guided to look at nine different locations on faces from the eyes to the mouth, including typical feature locations such as eyes, nose, and mouth, and some other atypical locations such as the cheeks and the corners of the mouth. In the study of Peterson and Eckstein (2012, 2013), participants had four fixation locations arranged vertically from the forehead to the middle of the eyes to the nose and to the mouth. Therefore, these previous studies tended to combine typical and atypical feature locations, resulting in performance that was influenced by atypical fixation locations. In contrast, in the current study, we only focused on two information‐rich fixation locations, on the eyes and the nose, where people generally prefer to look. When focusing on critical feature locations, our current study extends previous findings and suggests that the individuals' looking preferences are crucial for upper‐focused individuals but not for lower‐focused individuals to optimize face identification performance.

In addition to behavioral results, ERPs also showed that the P1‐to‐N170 peak‐to‐peak amplitude was larger when fixations were to the eyes rather than to the nose for the upper‐focused group only after correcting for the P1 baseline. For the lower‐focused group, the amplitude was similar between fixation conditions. This result is partially consistent with previous studies that found that the N170 or the P1‐to‐N170 peak‐to‐peak amplitude was larger for fixations on the eyes than the nose or mouth (De Lissa et al. 2014; McPartland et al. 2010; Nemrodov et al. 2014). Previous studies have found that the eyes are particularly salient components of faces and receive most fixations from observers (Royer et al. 2018; Schyns et al. 2002; Vinette et al. 2004). The presentation of just the eyes could activate face sensitive cells in the inferior temporal cortex almost as well as full faces, but face images without the eyes delayed the cell responses in that region for monkeys (Issa and DiCarlo 2012). Therefore, it appears that the N170 is especially sensitive to fixations onto the eyes (Nemrodov et al. 2014). However, this argument does not take differences in EM patterns between observers into account. Separating individuals by EM patterns, we have shown in this study that the neural responses were more sensitive to fixation on the eyes than the nose for upper‐focused observers but not for lower‐focused observers. Our findings indicate that the larger face‐sensitive components triggered by fixating on the eyes than on the nose are dependent upon individuals' looking preferences. The N170 can therefore be considered as an index that is influenced not just by looking position on faces but also by an individual's looking preference.

Given the significance of the eyes, one may think that people will look at the eyes as the entry point for optimally activating face perception and recognition processes. Indeed, many individuals (those who are upper‐focused observers) do look mostly at the eyes, and they show better identification performance and stronger neural activity when fixating on the eyes than the nose. However, there are also lower‐focused observers, who do not fixate on the eyes but instead look at the nose and mouth more frequently. They should have more experience directly fixating on the nose and mouth than on the eyes, and therefore it is not surprising that they identify faces well when looking at the nose, with corresponding activity triggered in face‐sensitive regions of the visual system. However, since the eyes have rich identity information, lower‐focused observers can also perform well when viewing them, resulting in equivalent performance when fixating either the eyes or the nose during identification, even though observers do not have the equivalent experience with them.

Other studies have also reported that the eye region is critical for upper‐focused observers. For example, Zhong et al. (2024) found that upper‐focused observers had a larger composite effect when judging upper halves of faces than judging lower halves of faces, whereas lower‐focused observers had a comparable composite effect no matter whether judging upper halves or lower halves. Zhong et al. argued that looking at the eye region might allow upper‐focused individuals to extract information from a larger visual field than when they fixate on other regions. Chakravarthula and Eckstein (2024), using a face adaptation aftereffect (FAE) paradigm, found that for upper‐focused observers the FAE could transfer to the lower fixation location when initially adapted with fixation on the eyes but not the opposite way, whereas the FAE did not transfer in either way for the lower‐focused observers. These findings provide further evidence that looking at the eyes may activate a larger facial perceptual field for upper‐focused observers.

Interestingly, we found that the P1 was larger when the initial fixation was on the nose than on the eye for both the upper‐focused group and the lower‐focused group. This finding is consistent with the findings of Peterson et al. (2014), but it is inconsistent with our prediction, as the P1 is considered as an indicator of low‐level visual cues such as luminance and contrast (Tanskanen et al. 2005; Tobimatsu and Celesia 2006), and previous studies did not find differences on the P1 among fixation locations (De Lissa et al. 2014; Nemrodov et al. 2014). One possibility is that those previous studies focused on the N170, so that electrodes with the largest negative N170 amplitudes, which were typically at parietal (P7/P8) or parieto‐occipital (PO7/PO8) electrodes, were selected for analysis in their studies. However, the P1 at those regions were relatively weak, since the effect of fixation location on the P1 occurred at the occipital region (Oz). Indeed, a previous study also found that the P1 was larger for fixations to lower halves of faces than to upper halves of faces (Zerouali et al. 2013). In addition, some previous studies found that the P1 was bigger for faces depicted with low spatial frequencies than those with high spatial frequencies, suggesting that the P1 reflects coarse/global processing of faces (Jeantet et al. 2019; Nakashima et al. 2008; Obayashi et al. 2009). In the current study, when the fixation was on the nose, participants might be primed to adopt a more global processing style compared to when the fixation was on the eyes. According to this logic, the P1 would be larger when fixations were on the nose.

Additionally, we found that the lower‐focused group showed a larger P1 amplitude difference between the nose and the eye fixation conditions than the upper‐focused group. These results imply that EM patterns modulate the effect of fixation location on the P1 as well. Lower‐focused individuals who tend to look at the center or the lower half of a face may use a more global processing strategy compared to upper‐focused individuals. Therefore, fixating on lower halves of faces may activate coarse/global processing more strongly than fixating on upper halves of faces for the lower‐focused group compared with the upper‐focused group, so that a bigger P1 amplitude difference is elicited. However, these P1 differences in activation of facial processing do not affect identification performance. This issue will also need to be addressed by future research.

In our study, fixations were concentrated on the middle of the eyes or the nose, as shown in Figure 2a. These results are consistent with the findings of Peterson and Eckstein (2012, 2013), who found that the individuals' landing positions on faces were around the vertical midline. In contrast, Arizpe et al. (2017) reported four EM pattern clusters, with fixations concentrated on the left eye, the right eye, the middle of the eyes, or the nose. The inconsistency between studies may relate to differences in viewing duration. In the study of Arizpe et al. (2017), participants viewed faces for up to 10 s. Such long durations for encoding allowed participants to explore facial features more widely compared with in our task, and thus their eye movements were able to be clustered into four groups. It may further indicate that eye movements on faces at early or late stages have different functions. For example, early fixations are primarily for rapid face identification, whereas later fixations are more useful for social interactions (Peterson and Eckstein 2012). In addition, the current study allocated participants' EM patterns into groups based on the ROIs and the transitions between ROIs. In contrast, Arizpe et al. (2017) clustered participants' eye movements based on peak density. These differences in methodology may contribute to the differences in clustering patterns.

In summary, we explored face identification performance of individuals adopting different EM patterns modulated by fixation location and related ERPs. Results revealed that the upper‐focused group performed better and also showed a larger P1‐to‐N170 peak‐to‐peak amplitude when they fixated on the eyes compared with the nose. In contrast, the lower‐focused group performed equally well and showed similar magnitudes of P1‐to‐N170 peak‐to‐peak amplitude between fixation locations. These results suggest that face identification performance is associated with the EM pattern and the face region that is being fixated. Face identification seems to be tuned by individual differences in preferred viewing locations, and the P1‐to‐N170 peak‐to‐peak amplitude reflects identification performance.

## Author Contributions

**Nianzeng Zhong:** conceptualization, methodology, software, data curation, investigation, formal analysis, writing – original draft, writing – review and editing. **Janet H. Hsiao:** conceptualization, methodology, software, writing – review and editing. **Antoni B. Chan:** methodology, formal analysis, software. **William G. Hayward:** writing – review and editing, conceptualization, methodology, supervision, funding acquisition, project administration, resources.

## Funding

This research was supported by the General Research Fund of the Hong Kong Research Grants Council to WGH (LU13605523 and LU13610225) and JH (17608621).

## Conflicts of Interest

The authors declare no conflicts of interest.

## Supporting information


**Data S1:** Supporting Information.

## Data Availability

The data and analysis scripts that support the findings of this study will be available after the manuscript gets accepted by the journal.

## Endnotes

^1^
The results for the cluster of Oz/O1/O2 channels, P7/P8 pair, and PO7/PO8 pair are in Part 1 of the Supporting Information.

^2^
When we collected data for Experiment 2, we used an older version of the EMHMM to generate representative patterns and compared the individuals' eye movements in Experiment 2 to those patterns to reach 23 participants in each group. After we finished the data collection, we adopted a more recent Bayesian approach to generate representative patterns which were the representative patterns in Experiment 1. The new patterns were slightly different from the previous patterns, and therefore, the individuals in subgroups were slightly different from previous. Eventually, we had 24 participants in the upper‐focused group and 22 participants in the lower‐focused group. None of the results between the groups using the new clustering method were meaningfully different from those found using the previous method.
