# Supplementary material for: Idiosyncratic Eye‐Movement Patterns to Faces Are Associated With Differences in Neural Processing: Evidence From Behavioral and Event‐Related Potential Responses
Source: Eur J Neurosci. 2026 Oct 22;64(8):e70726. doi: 10.1111/ejn.70726 (PMC13690617; doi:10.1111/ejn.70726)
Supplement: Supplementary file 1 [file EJN-64-0-s001.docx]

**Part 1**

In the main manuscript, we reported the statistical results for the P1 at the Oz and the N170 at the P8, where the most activated electrodes for the target ERPs are. For completeness, we reported the P1 at Oz, O1, and O2 clustering, and the N170 at the P7/P8 pair and the PO7/PO8 pair in the Supplementary1 below.

**1| ERPs in Experiment 1**

**1.1| ERPs in the one-fixation block**

For the P1 amplitude at the cluster of Oz, O1, and O2 channels (see Figure.1a), a 2 (Initial fixation location: eye vs. nose) × 2 (EM pattern: upper-focused vs. lower-focused) mixed ANOVA was conducted. The main effect of EM pattern was not significant, F (1,44) = 2.49, p = .12, *η_p_^2^* = .05. The main effect of initial fixation location was significant, F (1,44) = 47.56, p < .001, *η_p_^2^* = .52. The interaction effect was significant, F (1,44) = 7.59, p = .009, *η_p_^2^* = .15. Post hoc t-tests revealed that P1 amplitude was larger for the nose fixation condition than the eye fixation condition for both the lower-focused group (t (44) = 6.08, p < .001) and the upper-focused group (t (44) = 3.41, p = .008). An independent-samples t-test on the P1 difference for the upper-focused and the lower-focused groups revealed that the upper-focused group had smaller P1 amplitude difference than the lower-focused group (Mean: 1.16 vs 2.71, t (44) = -2.75, p = .009, cohen’s *d* = -0.84).

Figure. 1. (a) and (b) are averaged waveforms at Oz/O1/O2 channels in the one-fixation block and two-fixation block, respectively.

For peak-to-peak analysis on P1-to-N170 amplitude at P7/P8 channels (see Figure.2a), a 2 (Initial fixation location: eye vs. nose) × 2 (EM pattern: upper-focused vs. lower-focused) × 2 (hemisphere: left vs. right) mixed ANOVA was conducted. The main effect of initial fixation location was significant, F (1,44) = 7.40, p = .009, *η_p_^2^* = .14. The main effect of hemisphere was significant, F (1,44) = 33.06, p < .001, *η_p_^2^* = .43. The interaction between fixation location and EM pattern was significant, F (1,44) = 7.48, p = .009, *η_p_^2^* = .15. Post hoc t-tests revealed that the amplitude was larger in the eye fixation condition than in the nose fixation condition for the upper-focused group (t (44) = 4.49, p < .001), but no significant difference was found between fixation conditions for the lower-focused group, t (44) =-0.01, p > .05. No other significant effects were found (*p*_s_ >.05).

For peak-to-peak analysis on P1-to-N170 amplitude at PO7/PO8 channels, a 2 (Initial fixation location: eye vs. nose) × 2 (EM pattern: upper-focused vs. lower-focused) × 2 (hemisphere: left vs. right) mixed ANOVA was conducted. The main effect of hemisphere was significant, F (1,44) = 18.90, p < .001, *η_p_^2^* = .30. The interaction between fixation location and EM pattern was significant, F (1,44) = 12.46, p < .001, *η_p_^2^* = .22. Post hoc t-tests revealed that the amplitude was larger in the eye fixation condition than in the nose fixation condition for the upper-focused group (t (44) = 3.82, p = .002), but no significant difference was found between fixation conditions for the lower-focused group, t (44) =-1.52, p > .05. No other significant effects were found (*p*_s_ >.05).

Figure. 2. (a) and (b) are waveforms at P7/P8 pair and PO7/PO8 pair in the one-fixation block and two-fixation block, respectively.

**1.2| ERPs in the two-fixation block**

For the P1 amplitude at the cluster of Oz, O1, and O2 channels (see Figure.1b), a 2 (Initial fixation location: eye vs. nose) × 2 (EM pattern: upper-focused vs. lower-focused) mixed ANOVA was conducted. The main effect of EM pattern was not significant, F (1,47) = 1.26, p = .27, *η_p_^2^* = .03. The main effect of initial fixation location was significant, F (1,47) = 26.61, p < .001, *η_p_^2^* = .36. The interaction effect was significant, F (1,47) = 4.80, p = .03, *η_p_^2^* = .09. Post hoc t-tests revealed that P1 amplitude was larger for the nose fixation condition than the eye fixation condition for the lower-focused group (t (47) = 4.70, p < .001) but not for the upper-focused group (t (47) = 2.38, p >.05).

For peak-to-peak analysis on P1-to-N170 amplitude at P7/P8 channels (see Figure.2b), a 2 (Initial fixation location: eye vs. nose) × 2 (EM pattern: upper-focused vs. lower-focused) × 2 (hemisphere: left vs. right) mixed ANOVA was conducted. The main effect of initial fixation location was significant, F (1,47) = 14.75, p < .001, *η_p_^2^* = .24. The main effect of hemisphere was significant, F (1,47) = 38.59, p < .001, *η_p_^2^* = .45. The interaction between fixation location and EM pattern was marginal significant, F (1,47) = 3.64, p = .06, *η_p_^2^* = .07. Post hoc t-tests revealed that the amplitude was larger in the eye fixation condition than in the nose fixation condition for the upper-focused group (t (47) = 4.62, p < .001), but no significant difference was found between fixation conditions for the lower-focused group, t (47) =1.24, p > .05. No other significant effects were found (*p*_s_ >.05).

For peak-to-peak analysis on P1-to-N170 amplitude at PO7/PO8 channels, a 2 (Initial fixation location: eye vs. nose) × 2 (EM pattern: upper-focused vs. lower-focused) × 2 (hemisphere: left vs. right) mixed ANOVA was conducted. The main effect of hemisphere was significant, F (1,47) = 18.99, p < .001, *η_p_^2^* = .29. The interaction between fixation location and EM pattern was significant, F (1,47) = 12.55, p < .001, *η_p_^2^* = .21. Post hoc t-tests revealed that the amplitude was larger in the eye fixation condition than in the nose fixation condition for the upper-focused group (t (47) = 4.10, p < .001), but no significant difference was found between fixation conditions for the lower-focused group, t (47) =-1.27, p > .05. No other significant effects were found (*p*_s_ >.05).

**2| ERPs in Experiment 2**

**2.1| ERPs in the 500ms block**

For the P1 amplitude at the cluster of Oz, O1, and O2 channels (see Figure.3a), a 2 (Initial fixation location: eye vs. nose) × 2 (EM pattern: upper-focused vs. lower-focused) mixed ANOVA was conducted. The main effect of EM pattern was not significant, F (1,35) = 0.52, p = .47, *η_p_^2^* = .02. The main effect of initial fixation location was significant, F (1,35) = 20.38, p < .001, *η_p_^2^* = .37. The interaction effect was marginally significant, F (1,35) = 2.09, p = .097, *η_p_^2^* = .08. Post hoc t-tests revealed that P1 amplitude was larger for the nose fixation condition than the eye fixation condition for both the lower-focused group (t (35) = 4.34, p < .001), but not for the upper-focused group (t (35) = 2.02, p > .05).

Figure. 3. (a) and (b) are averaged waveforms at Oz/O1/O2 channels in the 500ms block and 250ms block, respectively.

For peak-to-peak analysis on P1-to-N170 amplitude at P7/P8 channels (see Figure.4a), a 2 (Initial fixation location: eye vs. nose) × 2 (EM pattern: upper-focused vs. lower-focused) × 2 (hemisphere: left vs. right) mixed ANOVA was conducted. The main effect of initial fixation location was significant, F (1,35) = 43.18, p < .001, *η_p_^2^* = .16. The main effect of hemisphere was significant, F (1,35) = 24.42, p < .001, *η_p_^2^* = .41. The interaction between fixation location and hemisphere was significant, F (1,35) = 7.93, p = .008, *η_p_^2^* = .19. The three-way interaction was significant, F (1,35) = 7.56, p = .009, *η_p_^2^* = .18. The interaction between fixation location and EM pattern was significant, F (1,35) = 6.77, p = .014, *η_p_^2^* = .16. Post hoc t-tests revealed that the amplitude was larger in the eye fixation condition than in the nose fixation condition for the upper-focused group (t (35) = 6.58, p < .001), and marginal significant difference was found between fixation conditions for the lower-focused group, t (35) =2.77, p = .053. No other significant effects were found (*p*_s_ >.05).

For peak-to-peak analysis on P1-to-N170 amplitude at PO7/PO8 channels, a 2 (Initial fixation location: eye vs. nose) × 2 (EM pattern: upper-focused vs. lower-focused) × 2 (hemisphere: left vs. right) mixed ANOVA was conducted. The main effect of initial fixation location was significant, F (1,35) = 29.10, p < .001, *η_p_^2^* = .45. The main effect of hemisphere was significant, F (1,35) = 24.86, p < .001, *η_p_^2^* = .42. The interaction between fixation location and hemisphere was significant, F (1,35) = 8.48, p = .006, *η_p_^2^* = .20. The three-way interaction was significant, F (1,35) = 8.85, p = .005, *η_p_^2^* = .20. The interaction between fixation location and EM pattern was significant, F (1,35) = 5.17, p = .029, *η_p_^2^* = .13. Post hoc t-tests revealed that the amplitude was larger in the eye fixation condition than in the nose fixation condition for the upper-focused group (t (35) = 5.50, p < .001), but no significant difference was found between fixation conditions for the lower-focused group, t (35) =2.18, p >.05. No other significant effects were found (*p*_s_ >.05).

Figure. 4. (a) and (b) are waveforms at P7/P8 pair and PO7/PO8 pair in the 500ms block and 250ms block, respectively.

**2.2| ERPs in the 250ms block**

For the P1 amplitude at the cluster of Oz, O1, and O2 channels (see Figure.3b), a 2 (Initial fixation location: eye vs. nose) × 2 (EM pattern: upper-focused vs. lower-focused) mixed ANOVA was conducted. The main effect of initial fixation location was significant, F (1,34) = 15.12, p < .001, *η_p_^2^* = .31. No other significant effects were found (*p*_s_ >.05).

For peak-to-peak analysis on P1-to-N170 amplitude at P7/P8 channels (see Figure.4b), a 2 (Initial fixation location: eye vs. nose) × 2 (EM pattern: upper-focused vs. lower-focused) × 2 (hemisphere: left vs. right) mixed ANOVA was conducted. The main effect of initial fixation location was significant, F (1,34) = 25.35, p < .001, *η_p_^2^* = .43. The main effect of hemisphere was significant, F (1,34) = 15.89, p < .001, *η_p_^2^* = .32. No other significant effects were found (*p*_s_ >.05).

For peak-to-peak analysis on P1-to-N170 amplitude at PO7/PO8 channels, a 2 (Initial fixation location: eye vs. nose) × 2 (EM pattern: upper-focused vs. lower-focused) × 2 (hemisphere: left vs. right) mixed ANOVA was conducted. The main effect of initial fixation location was significant, F (1,34) = 9.82, p = .004, *η_p_^2^* = .22. The main effect of hemisphere was significant, F (1,34) = 24.86, p < .001, *η_p_^2^* = .33. No other significant effects were found (*p*_s_ >.05).

**Part 2**

In the main manuscript, a few participants’ U-L scale values were close to zero, which means that their eye-movement patterns were in between the upper-focused and the lower-focused patterns. Here, we used an MAD (Median Absolute Deviation) -based method to set a boundary for near-zero threshold, and we re-analyzed the data excluding those participants whose U-L scale values were within the boundary.

**1| Near‑zero threshold based on robust dispersion estimate in Experiment 1**

For MAD-based threshold, we first calculate the median absolute deviation (MAD) of the data and then obtained the robust estimate of the standard deviation (σ_MAD) by multiplying the MAD by the constant 1.4826 (Rousseeuw & Croux, 1993). The near-zero threshold (τ) was defined as K × σ_MAD, with K = 0.025. Those UL-scale values whose were smaller than |τ| were excluded from subsequent analyses. In total, 10 participants (5 from the upper-focused group and 5 from the lower-focused group) were excluded. Therefore, there were 40 participants (26 from the upper-focused group and 14 from the lower-focused group) left for subsequent analyses.

**1.1| Behavioural Performance**

For the face identification performance between the upper-focused and lower-focused groups in the free EM block, an independent t-test revealed no significant difference of accuracy between EM pattern groups, t (38) = 0.42, p = .67, cohen’s *d* = 0.14 (see Figure.1).

For the face identification performance between groups in the one-fixation block, a 2 (Initial fixation location: eye vs. nose) × 2 (EM pattern: upper-focused vs. lower-focused) mixed ANOVA was conducted on accuracy. The main effect of initial fixation location was significant, F (1,38) = 6.23, p = .017, *η_p_^2^* = .14. The main effect of EM pattern was significant, F (1,38) = 4.25, p = .046, *η_p_^2^* = .10. The interaction effect was significant, F (1,38) = 23.15, p < .001, *η_p_^2^* = .38. Post hoc t-tests of this interaction revealed that the upper-focused group had higher identification accuracy for the eye condition than the nose condition, t (38) = 6.18, p < .001, while the lower-focused group had comparable identification accuracy between the eye and the nose conditions, t (48) = -1.44, p >.05.

For fixation duration in the one-fixation block, neither of the main effects of initial fixation location or EM pattern were significant, F (1,38) = 0.36, p = .55, *η_p_^2^* = .01 and F (1,38) = 0.24, p = .63, *η_p_^2^* = .01, respectively. The interaction effect was significant, F (1,38) = 15.20, p < .001, *η_p_^2^* = .29. Post hoc t-tests of this interaction revealed that the upper-focused group had longer fixation durations for the eye condition than the nose condition t (38) = 3.80, p = .003, while the lower-focused group had comparable fixation durations between the eye and the nose conditions, t (38) = -2.04, p >.05.

For the face identification performance between groups in the two-fixation block, the main effects of initial fixation location and EM pattern were not significant, F (1,38) = 0.00, p = .99, *η_p_^2^* = .00 and F (1,38) = 0.09, p = .76, *η_p_^2^* = .002, respectively. The interaction between initial fixation location and EM pattern was significant, F (1,38) = 11.7, p = .001, *η_p_^2^* = .24. Post hoc t-tests revealed that the upper-focused group had significant better performance on the eye condition than the nose condition, t (38) = 2.88, p = .04, while the lower-focused group had comparable performance on the eye and the nose conditions, t (38) = -2.13, p >.05.

For the first fixation duration in the two-fixation block, the main effects of initial fixation location and EM pattern were not significant, F (1,38) = 0.28, p = .60, *η_p_^2^* = .01 and F (1,38) = 0.12, p = .73, *η_p_^2^* = .001, respectively. The interaction effect was significant, F (1,38) = 23.39, p < .001, *η_p_^2^* = .38. Post hoc analysis of this interaction revealed that participants in the upper-focused group had longer fixation durations for the eye condition than the nose condition t (38) = 4.54, p < .001, while those in the lower-focused group had comparable fixation durations between the eye and the nose conditions, t (48) = -2.67, p >.05.

For the total fixation duration, the main effect of initial fixation location was significant, F (1,38) = 4.62, p = .04, *η_p_^2^* = .11. The main effect of EM pattern was not significant, F (1,38) = 0.02, p = .90, *η_p_^2^* = .000. The interaction effect was significant, F (1,48) = 15.31, p < .001, *η_p_^2^* = .24. Post hoc analysis of this interaction revealed that the upper-focused group had longer fixation duration for the eye condition than the nose condition t (38) = 5.31, p < .001, while the lower-focused group had comparable duration between the eye and the nose conditions, t (38) = -1.23, p >.05.

Figure. 1. (a) Accuracy in the free eye-movement block; (b) and (c) Accuracy and fixation duration in the one-fixation block and two-fixation blocks, respectively.

**1.2| ERP components in the one-fixation block**

Four participants were further excluded for ERP analysis in this block because of high data noise. Therefore, thirty-six participants (23 in the upper-focused group and 13 in the lower-focused group) remained(see Figure.2).

For P1 amplitude, a 2 (Initial fixation location: eye vs. nose) × 2 (EM pattern: upper-focused vs. lower-focused) mixed ANOVA was conducted. The main effect of EM pattern was not significant, F (1,34) = 0.20, p = .65, *η_p_^2^* = .01. The main effect of initial fixation location was significant, F (1,34) = 31.73, p < .001, *η_p_^2^* = .48. The interaction effect was significant, F (1,34) = 5.62, p = .02, *η_p_^2^* = .14. Post hoc t-tests revealed that P1 amplitude was larger for the nose fixation condition than the eye fixation condition for the lower-focused group (t (34) = 5.01, p < .001) and marginally significant for the upper-focused group (t (34) = 2.72, p = .06). An independent-samples t-test on the P1 difference for the upper-focused and the lower-focused groups revealed that the upper-focused group had smaller P1 amplitude difference than the lower-focused group (Mean: 1.26 vs 3.10, t (34) = -2.37, p = .02, cohen’s *d* = -0.82).

For P1 latency, the main effect of initial fixation location was significant, F (1,34) = 10.50, p =.003, *η_p_^2^* = .24. No other significant effects were found for P1 latency (*p*_s_ >.05).

For peak-to-peak analysis on P1-to-N170 amplitude, the interaction between fixation location and EM pattern was significant, F (1,34) = 5.24, p = .03, *η_p_^2^* = .13. Post hoc t-tests revealed that the amplitude was larger in the eye fixation condition than in the nose fixation condition for the upper-focused group (t (34) = 2.87, p = .04), but no significant difference was found between fixation conditions for the lower-focused group, t (34) =-0.71, p > .05. No other significant effects were found (*p*_s_ >.05).

Figure. 2. The P1 amplitudes at Oz and the P1-to-N170 amplitudes at P8 in bar graphs in one-fixation block and two-fixation block, respectively.

**1.3| ERP components in the two-fixation block**

One participant was further excluded for ERP analysis in this block because of high data noise. Therefore, thirty-nine participants (25 in the upper-focused group and 14 in the lower-focused group) remained(see Figure.2).

For P1 amplitude, the main effect of initial fixation location was significant, F (1,37) = 36.93, p < .001, *η_p_^2^* = .50. The interaction effect was significant, F (1,37) = 7.31, p = .01, *η_p_^2^* = .17. Post hoc t-tests revealed that P1 amplitude was larger for the nose fixation condition than the eye fixation condition for both the upper-focused group (t (37) = 2.82, p = .008) and the lower-focused group (t (37) = 5.48, p < .001). The independent-samples t-test on the P1 difference between fixation conditions for the upper-focused and the lower-focused groups revealed that the upper-focused group had smaller P1 amplitude difference than the lower-focused group (Mean: 1.23 vs 3.21, t (37) = -2.70, p = .01, cohen’s *d* = 0.90. See Fig. 5).

For P1 latency, the main effect of initial fixation location was significant, F (1,37) = 17.08, p < .001, *η_p_^2^* = .32. No other significant effects were found (*p*_s_ >.05).

For peak-to-peak analysis on P1-to-N170 amplitude, the main effect of initial fixation location was significant, F (1,37) = 6.04, p = .019, *η_p_^2^* = .14. The interaction between fixation location and EM pattern was marginal significant, F (1,37) = 3.40, p = .073, *η_p_^2^* = .08. Post hoc t-tests revealed that the P1-to-N170 amplitude was larger in the eye fixation condition than in the nose fixation condition for the upper-focused group (t (37) = 3.59, p = .006), but no significant difference was found between fixation conditions for the lower-focused group, t (37) =0.38, p > .05.

**2| Near‑zero threshold based on robust dispersion estimate in Experiment 2**

Using the same MAD measurement as Experiment 1, 9 participants (3 from the upper-focused group and 6 from the lower-focused group) were excluded in Experiment 2. Therefore, there were 37 participants (21 from the upper-focused group and 16 from the lower-focused group) left for subsequent analyses.

**2.1| Behavioural Performance**

For the face identification performance between the upper-focused and lower-focused groups in the free EM block, an independent t-test revealed no significant difference of accuracy between EM pattern groups, t (35) = 1.00, p = .32, cohen’s *d* = 0.33 (see Figure.3a).

In the 250ms duration block, a 2 (Initial fixation location: eye vs. nose) × 2 (EM pattern: upper-focused vs. lower-focused) mixed ANOVA was conducted on accuracy. The main effects of initial fixation location and EM pattern were not significant, F (1,35) = 0.21, *p* = .65, *η_p_^2^* = .006, and F (1,35) = 1.26, *p* = .27, *η_p_^2^* = .04, respectively. The interaction between fixation location and EM pattern was significant, F (1,35) = 13.96, *p* <.001, *η_p_^2^* = .29. Post hoc t-tests of this interaction revealed that the upper-focused group had higher identification accuracy for the eye condition than the nose condition, t (35) = 3.19, *p* = .02, while the lower-focused group did not show differences between the eye and the nose conditions, t (35) = -2.06, *p* >.05.

In the 500ms duration block, the main effect of initial fixation location and EM pattern were not significant, F (1,35) = 1.51, *p* = .23, *η_p_^2^* = .04, and F (1,35) = 2.66, *p* = .11, *η_p_^2^* = .07, respectively. The interaction between fixation location and EM pattern was significant, F (1,35) = 17.13, *p* < .001, *η_p_^2^* = .33. Post hoc t-tests of this interaction revealed that the upper-focused group had higher identification accuracy for the eye condition than for the nose condition, t (35) = 4.08, *p* = .001, while the lower-focused group did not show differences between the eye and the nose conditions, t (44) = -1.93, *p* >.05.

To compare the behavioral performance between 250ms and 500ms blocks, a 2 (Initial fixation location: eye vs. nose) × 2 (Fixation duration: 250ms vs. 500ms) × 2 (EM pattern: upper-focused vs. lower-focused) 3-way mixed ANOVA was conducted on accuracy. Only the interaction effect between fixation location and EM pattern was significant, F (1,35) = 27.03, *p* <.001, *η_p_^2^* = .44. No other effects were significant (*p*_s_ > .05), suggesting that fixation duration did not modulate identification performance in this experiment.

Figure.3. (a) Accuracy in the free eye-movement block, 500ms block, and 250ms block, respectively; (b) and (c) The P1 amplitudes at Oz and the P1-to-N170 amplitudes at P8 in bar graphs in 500ms block and 250ms block, respectively.

**1.2| ERP components in the 500ms block**

Seven participants were excluded for ERP analysis in this block because of high data noise. Therefore, thirty participants (18 in the upper-focused group and 12 in the lower-focused group) remained (see Figure.3b).

For P1 amplitude, a 2 (Initial fixation location: eye vs. nose) × 2 (EM pattern: upper-focused vs. lower-focused) mixed ANOVA was conducted. The main effect of initial fixation location was significant, F (1,28) = 23.35, p < .001, *η_p_^2^* = .46. The interaction effect was significant, F (1,28) = 5.16, p = .03, *η_p_^2^* = .16. Post hoc t-tests revealed that P1 amplitude was larger for the nose fixation condition than the eye fixation condition for the lower-focused group (t (28) = 4.59, p < .001) but showed no differences between fixation conditions for the upper-focused group (t (28) = 2.02, p > .05). No other effects were significant (*p*_s_ > .05).

For P1 latency, the main effect of initial fixation location was significant, F (1,28) = 5.26, p = .03, *η_p_^2^* = .16. No other significant effects were found for P1 latency (*p*_s_ >.05).

For N170 amplitude, the main effect of initial fixation location was significant, F (1,28) = 28.24, p <.001, *η_p_^2^* = .50. The interaction between fixation location and EM pattern was significant, F (1,28) = 4.52, p = .042, *η_p_^2^* = .14. Post hoc t-tests revealed that the N170 was larger in the eye fixation condition than in the nose fixation condition for the upper-focused group (t (28) = 5.89, p <.001) but not for the lower-focused group, t (28) =2.06, p > .05. No other significant effects were found (*p*_s_ >.05).

For peak-to-peak analysis on P1-to-N170 amplitude, the main effect of initial fixation location was significant, F (1,28) = 19.91, p <.001, *η_p_^2^* = .42. The interaction between fixation location and EM pattern was significant, F (1,28) = 8.24, p = .008, *η_p_^2^* = .23. Post hoc t-tests revealed that the amplitude was larger in the eye fixation condition than in the nose fixation condition for the upper-focused group (t (28) = 5.80, p <.001), but no significant difference was found between fixation conditions for the lower-focused group, t (28) =1.03, p > .05. No other significant effects were found (*p*_s_ >.05).

**1.2| ERP components in the 250ms block**

Eight participants were excluded for ERP analysis in this block because of high data noise. Therefore, twenty-nine participants (17 in the upper-focused group and 12 in the lower-focused group) remained (see Figure.3c).

For P1 amplitude, a 2 (Initial fixation location: eye vs. nose) × 2 (EM pattern: upper-focused vs. lower-focused) mixed ANOVA was conducted. The main effect of initial fixation location was significant, F (1,27) = 18.29, p < .001, *η_p_^2^* = .40. No other effects were significant (*p*_s_ > .05).

For P1 latency, the main effect of initial fixation location was significant, F (1,27) = 10.66, p = .003, *η_p_^2^* = .28. No other significant effects were found for P1 latency (*p*_s_ >.05).

For N170 amplitude, the main effect of initial fixation location was significant, F (1,27) = 11.25, p =.002, *η_p_^2^* = .29. No other significant effects were found (*p*_s_ >.05).

For peak-to-peak analysis on P1-to-N170 amplitude, the main effect of initial fixation location was significant, F (1,27) = 18.43, p <.001, *η_p_^2^* = .41. No other significant effects were found (*p*_s_ >.05).

**Reference:**

Rousseeuw, P. J., & Croux, C. (1993). Alternatives to the median absolute deviation. Journal of the American Statistical association, 88(424), 1273-1283.

**Part 3**

In the main manuscript, we reported that the optimal number of representative patterns was two. Here, we showed the models’ log-likelihoods for K from 1 to 6 to justify the determination.
